# ‘Not my mess’?: How do supporters of individuals with hoarding difficulties rate the quality of the support they offer?

**DOI:** 10.1111/bjc.12520

**Published:** 2024-11-22

**Authors:** James Dennis, Kate Rosen, Paul M Salkovskis

**Affiliations:** ^1^ University of Oxford Oxford UK; ^2^ Oxford Cognitive Therapy Centre Oxford UK; ^3^ Department of Experimental Psychology Oxford Health NHS Foundation Trust University of Oxford and Oxford Health NHS Foundation Trust Oxford UK

**Keywords:** hoarding, OCD, social support, stigmaassociative stigma

## Abstract

**Objectives:**

Hoarding difficulties (HD) affect many people and cause upset and danger for the person, as well as friends and family. Previous research found that people with HD feel less adequately socially supported compared with individuals with obsessive compulsive disorder (OCD). This study used the perspective of those offering support to infer whether people with HD view their support differently, or if there is a gap in support quality compared with those with OCD.

**Design:**

The design was cross‐sectional, comparing those supporting OCD with those supporting HD.

**Methods:**

An online questionnaire was completed by 116 people offering support (POS) to people with these conditions. Support quality was measured using an adapted, proxy version of the Revised Norbeck Social Support Questionnaire. The research hypothesized that POS(HD) would not differ on support ratings compared with POS(OCD); or that POS(HD) would report comparatively lower ratings across support components. Secondary analysis investigated group differences in stigmatized attitudes and associative stigma; internalized stigma by virtue of having a connection to a stigmatized individual.

**Results:**

POS(HD) rated their wish to support and the perceived success as significantly lower. Public stigma was rated more highly by POS(HD) relative to POS(OCD) and associative stigma felt more acutely by POS(HD).

**Conclusions:**

Motivation to support was significantly lower in the HD group with associative stigma a significant predictive factor. Further research involving dyads is needed to investigate what is causing this shortfall in support. Clinical research for HD interventions should also examine how involving POS could enhance treatment outcomes.

**Practitioner points**

- Individuals offering support to people with hoarding difficulties may find difficulty in maintaining a commitment to offering help which is known to lead to worse outcomes for the person with the condition.
- Clinicians should note that these supporters may have particular vulnerability to associative stigma and treatment for the person with the condition may be aided by helping supporters to acknowledge and resist their own stigma.
- In hoarding difficulties, supporters may be family members or partners, but there may also be vital help available from friends or other types of contacts.

## INTRODUCTION

Clinically significant hoarding difficulties (HD) were previously thought to be a symptom of obsessive compulsive disorder (OCD) or obsessive compulsive personality disorder (OCPD) (Gordon et al., 2013) and efforts to delineate HD from OCD continue. HD refers to:

Persistent difficulty discarding or parting with possessions, regardless of their actual value […] accompanied by an excessive acquisition of items that are not needed or for which there is no available space (American Psychiatric Association, 2013, p. 247).

Accumulated items include food waste, packaging, newspapers, animals, etc. resulting in obtrusive clutter (see Pertusa et al., 2019). The lifetime population prevalence of HD has been estimated at 2.6% from meta‐analysis, although this may be an underestimation due to barriers to seeking and accessing professional support (Postlethwaite et al., 2019).

Hoarding also impacts people who offer social support. Disruption and danger caused by clutter can become a burden for families and can result in the development of interpersonal conflict and even hostility (Tolin et al., 2008). Qualitative research conducted in the US (Sampson, 2013) and the UK (Wilbram et al., 2008) has offered insight into carers' guilt in light of their own problems understanding HD, feelings of hopelessness in the context of ineffective or absent professional support, and resentment at themselves becoming increasingly socially isolated as a result of support efforts. Sharing a home with a person that hoards can prompt family members to seek to escape the clutter, avoid the people‐with‐the‐conditions' (PWC) domestic space, and over time, become alienated from that relationship (Garrett, 2020). Relationships between people offering support (POS) and PWC appear to be most under threat when there is disagreement as to whether the PWC recognize the clutter as a problem (Büscher et al., 2014). Alternatively, supporters may become overwhelmed by the carer burden; a self‐perceived chronic and multifaceted strain on one's ability to provide support, arising from competing responsibility demands and exhausting emotional and material resources, resulting in decreased care provision and quality‐of‐life in the POS (Liu et al., 2020). Estrangement from POS may have catastrophic consequences as a lack of supportive relationships has been associated with overall poorer outcomes for the PWC (Chen et al., 2022).

POS to people with OCD are also noted to experience hopelessness, reduced quality‐of‐life, burden, and depressive symptoms (Cicek et al., 2013; Geffken et al., 2006). OCD social support research has tended to focus on the concept of accommodation, whereby family members of individuals with OCD perform well‐intentioned actions to alleviate distress through offering reassurance and/or facilitating rituals, but which are associated with increased OCD severity (La Buissonnière‐Ariza et al., 2022). Research by Kobori et al. (2017) found that specific forms of response to rituals may be difficult to maintain consistently, resulting in anxiety and frustration in the PWC. However, POS are often invited to offer reassurance in subtle ways, which are responded to gratefully by the PWC, establishing a predictable and expected helping role (Kobori et al., 2017). Research examining family support for 151 adults with OCD found that accommodation occurred at least weekly for 87% (La Buissonnière‐Ariza et al., 2022). In this context, family‐inclusive treatments for adults with OCD are emerging, with an early indication that family involvement may produce better outcomes than individual cognitive behavioural therapy (CBT) (Stewart et al., 2020). Accommodation is less well studied in HD populations; however, it is thought to occur at a similar rate to that of OCD (Drury et al., 2014; Vorstenbosch et al., 2015). Despite this, HD treatments involving POS are rare and have focussed on the POS' experience, rather than enhancing outcomes for the PWC (Thompson et al., 2017). Proof‐of‐concept studies indicate that support for supporters of HD is acceptable (Chasson et al., 2014) and increase positive experiences of caregiving (Thompson et al., 2016).

Maintaining supportive connections may be further adversely impacted by prevalent stigmatized attitudes towards individuals who hoard (Chasson et al., 2018), with emerging evidence of family members internalizing stigma through their association to the PWC (Prosser et al., 2024). Associative stigma–also referred to as courtesy stigma or affiliate stigma–was originally defined by Goffman (1963) and subsequent research has applied this concept to mental health stigma and investigated mediating factors, including cohabiting as a factor increasing internalized associative stigma (van der Sanden et al., 2015), as well as considering how POS might recognize and fight against stigma (Angermeyer et al., 2003). It is important to understand the extent to which such factors are specific to POS for HD.

It has been suggested that pre‐occupation with objects is a form of behavioural avoidance of navigating unpredictable early relationships and that attachment holds the key to improving HD treatment effectiveness (David et al., 2021; Kyrios et al., 2018; Medard & Kellett, 2014). However, attachment problems have been shown to be relevant but not specific to HD, relative to OCD. Barton et al. (2021) found that people with HD and people with OCD did not differ on measures of interpersonal attachment and relational needs, and these clinical groups had comparably smaller social networks than community controls. However, those with HD perceived comparatively less support from that network (Barton et al., 2021). Edwards et al. (2023) replicated this finding with a more rigorous measure, and PWC(HD) were again found to have similarly small social networks and rated the quality of support they received as significantly worse than the OCD and community control groups.

It could be simply a matter of *perception*; that is, those with HD have similar interactions with POS, but do not believe themselves to be supported. Alternatively, it could be that POS are actually responding more negatively to those with HD, due for example to particular attitudes and beliefs about hoarding, or how they view their own role as a supporter. To investigate the question of perception versus inadequate support, this study examines how POS perceive their efforts to support and how it is received.

### Hypotheses

The present research hypothesizes that the perceived poorer support in PWC could arise from two possible sources:

- Those with HD have similarly supportive interactions with POS, relative to PWC(OCD), but appraise the support they receive to be poorer.

- If this is the case, we would expect that POS(HD) will not differ in terms of their expressed wish to provide support and will report less successful support, compared with POS(OCD).

Or:

b POS are responding more negatively to those with HD, relative to OCD, being ambivalent about or disengaged from offering support.

- If this is the case, we would expect that POS(HD) will report a lower expressed wish to offer support and find their efforts to be less successful.

In both instances, the expectation is that POS(HD) will experience lower satisfaction in offering support, relative to POS(OCD). In line with literature describing estrangement, it is predicted that carer burden would be higher in the HD group, relative to POS(OCD), indicating an increased risk of POS becoming unable to continue support.

Secondary hypotheses were considered in relation to the impact stigma has on supportive interactions. It was hypothesized that HD would be rated as a more stigmatized condition compared with OCD and that public stigma presence would translate into internalized associative stigma, which would be reported more intensely by the POS(HD) group versus OCD counterparts.

## METHOD

### Overview

POS were sought via advertising on social media and sharing adverts via national OCD and HD charities and local support groups (materials in Data S1). Participants completed an online survey eliciting the frequency and types of support being offered, the experience of offering support, and its perceived effectiveness. No financial incentive was provided.

A power calculation using G*power (Faul et al., 2007), based on a mixed model ANOVA for the primary analysis, indicated a required sample size of 84. This was based on a type 2 error probability rate of 0.2 and an effect size of 0.25. In the absence of any similar previous studies, the effect size was conservatively estimated based on subscale differences of the Revised Norbeck Social Support Questionnaire (NSSQ‐R) between HD and OCD groups observed in the Edwards et al. (2023) study.

Ethical approval was granted by the University of Oxford Medical Sciences Interdivisional Research Ethics Committee, ref: R86617/RE001 (see Data S3).

### Participants

The concept of POS was operationalized by potential supporters endorsing that there was a person ‘that you have a personal connection to, e.g. a family member or friend you have known for a number of years, that you offer some form of support to’. The PWC were specified as having to be experiencing OCD or HD. To be eligible, POS and the PWC were 30+ years‐old and living in the UK. This age limit was selected to mitigate potential differences between the condition groups, with HD typically reaching clinically significant levels later in life (Zaboski II et al., 2019). A formal diagnosis was not required. Validity checks were used to confirm a clinically significant level of impact on the PWC' functioning. The Work and Social Adjustment Scale (WSAS) has been validated for use with OCD and depression and found to have good‐to‐excellent internal consistency (Mundt et al., 2002) and was adapted for POS proxy reporting. The Clutter Images Rating Scale (CIR) (Frost et al., 2008) was used to validate HD symptomology in the HD group. The CIR asks respondents to select images that best resemble the level of clutter in a person's home. The measure has good convergence with other measures of HD symptom severity and excellent inter‐rater reliability (Frost et al., 2008).

Given that OCD traits are exhibited by many people with HD, with diagnosable comorbidity understood to be around 18% (Pertusa et al., 2019), group designation for POS to individuals believed to have both OCD *and* HD was determined by POS identifying which they perceived to be the PWC' ‘main problem’.

### Measures

The full survey is included in Data S4. Demographic information was recorded as per potential relevance to the research hypotheses, for example, previous research has found consistent associations between age and HD prevalence (Postlethwaite et al., 2019).

#### Social support quality and experience

As no existing scale was identified, to measure POS' self‐rating of support quality a novel proxy scale was developed by inverting items from the NSSQ‐R (Norbeck et al., 1981), as well as developing additional items relevant to offering rather than receiving support. This proxy scale (P‐NSSQ‐R) offered a means of assessing differences on three subscales: wish to support, perceived support success, and impact of support on the POS. Existing items from the NSSQ‐R were transformed into two variants to distinguish between the POS's view of the level of effort they put into supportive actions and the perceived success of these efforts with regard to the observed PWC' response. Whereas an original NSSQ‐R item asked ‘how much do they make you feel liked or loved’, the wish to support subscale included items targeting intention and preparedness to support, such as ‘how much do you *try* to make the person feel liked or loved?’. Perceived support success assessed indicators of effectiveness of the support, such as ‘how much do you *actually* make this person feel liked or loved?’. Additional items were created following consultation with experts‐by‐experience with HD to aid construct validity by including ideas regarding conflict, frustration, and sense of purpose. For example, the P‐NSSQ‐R asks ‘does the person reject your help and support?’. Impact of support on the POS examined responses to and satisfaction taken from offering support, including ‘do you find helping and supporting the person rewarding?’

Higher P‐NSSQ‐R scores indicate more positive outcomes, that is, higher motivation to support, greater sense of the support helping the PWC, and less negative personal consequences experienced from offering support. Full items and scoring information for the P‐NSSQ‐R measure are included in Data S5.

16 items from the Burden Scale for Family Caregivers (BSFC) (Grau et al., 2015) were included to support the validity of the impact on supporter P‐NSSQ‐R subscale and to measure the burden in a broader sense, acknowledging supporters' resources and other responsibilities. The higher the total score, the greater the presence of carer burden and the increased likelihood of the caregiver developing mental health distress. A subset of the full 28‐item BSFC has been found to have good construct validity in that it showed a higher burden in supporters with less access to support resources and predicted future inpatient admission of the PWC on follow‐up (Graessel et al., 2014).

#### Stigma

For the secondary analysis, the measurement of both public stigma attitudes and associative stigma was necessary to delineate between POS' agreement with stigmatized ideas towards people with HD and OCD and the degree to which POS recognize themselves as a target of stigma by virtue of their connection to the stigmatized individual. Ten items from the Community Attitudes Towards the Mentally Ill Scale (CAMI) (Taylor & Dear, 1981) were used to assess the POS' general attitudes towards individuals with both OCD and HD. The CAMI is regularly used in research investigating prejudicial public attitudes and has been found to have well‐validated psychometric properties (Fox et al., 2018). A subset of the original 40 items was selected to avoid respondent fatigue. A 10‐item version of the CAMI was evaluated demonstrating good test–retest reliability (Sanabria‐Mazo et al., 2023), indicating appropriateness in using the scale for two separate targets of stigma consecutively. For this scale, higher scores indicated a higher overall presence of mental health stigma towards OCD and HD separately.

The 22‐item Affiliate Stigma Scale (ASS) (Mak & Cheung, 2008) measures the extent of self‐stigmatization of those who are associated with individuals with a mental illness. Three subscales are used to assess the associative stigma within affective, behavioural, and cognitive domains: the felt sense of stigma attempts to distance oneself from the stigmatized individual and beliefs about them and their condition. The validity of the ASS has been supported by consistently high internal consistency as well as total scale and item unidimensionality being supported by Rasch analysis (Chang et al., 2015). Higher scores on the ASS indicate a higher level of associative stigma.

### Analysis

The main analysis used a mixed model ANOVA assessing for significant differences between the HD and OCD groups using subscale scores on the P‐NSSQ‐R. For other variables, mixed model ANOVAs were used to compare groups on descriptive variables. Chi‐square analyses were used for categorical variables and independent‐ or paired‐samples *t*‐tests were used for follow‐up comparisons where main effects were identified via ANOVAs. Checks for normality of data were completed and test assumptions were met other than where specified. Where data were skewed, Mann–Whitney U tests were used for comparing mean differences in scale data. In line with the main hypothesis, two‐sided *p*‐values were used.

## RESULTS

Internal consistency of the P‐NSSQ‐R subscales and CAMI and BSFC measures were checked, with acceptable to excellent ratings (wish to support *α* = .83, perceived support success *α* = .94, impact of support on POS *α* = .83).

A total of 116 POS to somebody with OCD and/or hoarding difficulties completed the survey. Five cases from the HD group were removed as scores on the CIR did not indicate a clinically significant hoarding problem, leaving a final total of 111 cases. The HD group contained responses from 69 POS, with 19 of these PWC believed to have comorbid OCD. The OCD group contained responses from 42 POS, with 18 of these PWC believed to have comorbid HD. Of the PWC in the HD group, 29 (42.0%) were thought by the POS to self‐identify as having that condition compared with 39 (92.9%) in the OCD group.

Demographic information is presented in Table 1 (additional detail is available in Data S7). Group differences were assessed using Chi‐square tests, where significant, partitioned Chi‐square tests were used. The frequency of the PWC being family members versus friends, neighbours, or other types of relationship was not significantly different between the groups, *X* ^2^ (1, *N* = 80) = .042, *p* = .838. However, the OCD group had significantly more spouses or significant others versus family members compared with the HD group *X* ^2^ (1, *N* = 80) = 6.0, *p* = .014, and more spouses or significant others than friends and other types of relationships *X* ^2^ (1, *N* = 80) = 5.2, *p* = .023.

**TABLE 1:** Demographic Information.

	PWC			POS		
	HD	OCD	Total	HD	OCD	Total
	(*N* = 69)	(*N* = 42)	(*N* = 111)	(*N* = 69)	(*N* = 42)	(*N* = 111)
Age	*M* = 63.0, *SD* = 12.9	*M* = 41.2, *SD* = 10.7	*M* = 54.8, *SD* = 16.1	*M* = 52.2, *SD* = 13.4	*M* = 47.6, *SD* = 14.6	*M* = 50.4, *SD* = 14.0
Gender identity						
Woman	39 (56.5%)	21 (50.0%)	60 (54.1%)	59 (85.5%)	28 (66.7%)	87 (78.4%)
Man	28 (40.6%)	19 (45.2%)	47 (42.3%)	6 (8.7%)	12 (28.6%)	18 (16.2%)
Trans‐gender	2 (2.9%)	–	2 (1.8%)	3 (4.3%)	1 (2.4%)	4 (3.6%)
Non‐binary	–	2 (4.8%)	2 (1.8%)	–	–	–
Prefer not to say	–	–	–	1 (1.4%)	1 (2.4%)	2 (1.8%)
Ethnicity						
White British	59 (85.5%)	35 (83.3%)	94 (84.7%)	60 (87.0%)	33 (78.6%)	93 (83.8%)
Minority ethnic identity	10 (14.5%)	7 (16.7%)	17 (15.3%)	9 (13.0%)	9 (21.4%)	18 (16.2%)
Marital status						
Married or in a civil partnership	18 (26.1)	11 (26.2%)	29 (26.1%)	30 (43.5%)	18 (42.9%)	48 (43.2%)
Cohabiting	7 (10.1%)	8 (19.0%)	15 (13.5%)	15 (21.7%)	11 (26.2%)	26 (23.4%)
Divorced or separated	12 (17.4%)	1 (2.4%)	13 (11.7%)	8 (11.6%)	2 (4.8%)	10 (9.0%)
Widowed	7 (10.1%)	1 (2.4%)	8 (7.2%)	–	3 (7.1%)	3 (2.7%)
Single	25 (36.2%)	21 (50.0%)	46 (41.4%)	16 (23.2%)	8 (19.0%)	24 (21.6%)
Employment status						
Currently employed	22 (31.9%)	21 (50.0%)	43 (38.7%)	44 (63.8%)	27 (64.3%)	46 (41.4%)
Not currently employed	47 (68.1%)	21 (50.0%)	68 (61.3%)	25 (36.2%)	15 (35.7%)	8 (7.2%)

	HD (*N* = 69)	OCD (*N* = 42)	Total (*N* = 111)
Do the PWC have formal diagnoses?			
HD	6 (8.7%)	–	6 (5.4%)
OCD	3 (4.3%)	29 (69.0%)	32 (28.8%)
HD and OCD	–	1 (2.4%)	1 (.9%)
Neither HD nor OCD	60 (87.0%)	12 (28.6%)	72 (64.9%)
Have the PWC sought help for their condition?			
Yes	18 (26.1%)	31 (73.8%)	49 (44.1%)
No	51 (73.9%)	11 (26.2%)	62 (55.9%)

Years since onset of PWC' condition	*M* = 25.6, *SD* = 13.7	*M* = 22.2, *SD* = 11.9	*M* = 24.3, *SD* = 13.1
Relationship type to PWC			
Spouse or partner	8 (11.6%)	16 (38.1%)	24 (21.6%)
Significant other	5 (7.2%)	2 (4.8%)	7 (6.3%)
Family member or relative	36 (52.2)	16 (38.1%)	52 (46.8%)
Neighbour	1 (1.4%)	–	1 (.9%)
Friend	18 (26.1%)	5 (11.9%)	23 (20.7%)
Work associate	–	1 (2.4%)	1 (.9%)
Other	1 (1.4%)	2 (4.8%)	3 (2.7%)

Years known	*M* = 32.4, *SD* = 17.4	*M* = 22.5, *SD* = 16.4	*M* = 28.7, *SD* = 17.1
Do the POS live with the PWC?			
Yes	20 (29.0%)	25 (59.5%)	45 (40.5%)
No	49 (71.0%)	17 (40.5%)	66 (59.5%)
WSAS scaled total score^a^	*M* = 5.1, *SD* = 1.9	*M* = 4.8, *SD* = 2.0	*M* = 5.0, *SD* = 1.9
CIR total score	*M* = 16.1, *SD* = 4.9	*M* = 7.8, *SD* = 4.9	*M* = 12.9, *SD* = 6.3

^a^ WSAS scores divided by applicable items after considering if PWC are unemployed or retired for reasons other than their condition.

In the OCD group, PWC age data were skewed, so a Mann–Whitney *U* test was selected. PWC were significantly older in the HD group (*U* = 298, *p* < .001) and had known the POS for longer *t*_(109)_ = 3.0, *p* = .003. PWC more commonly had a formal diagnosis in the OCD group, *X* ^2^ (1, *N* = 111) = 39.1, *p* < .001, and were more likely to have sought help for their condition, *X* ^2^ (1, *N* = 111) = 24.1, *p* < .001. There were no significant differences for PWC between male and female gender identity, *X* ^2^ (1, *N* = 107) = .33, *p* = .565, nor White British versus minority ethnicity, *X* ^2^ (1, *N* = 111) = .10, *p* = .758. Similarly, there were no significant differences between groups when comparing whether PWC were currently in a relationship, *X* ^2^ (1, *N* = 111) = .89, *p* = .347, or were currently in paid employment versus not employed, *X* ^2^ (1, *N* = 111) = 3.6, *p* = .057.

POS' age in the OCD group was also skewed at the lower range. POS did not significantly differ in age between groups (*U* = 1143.5, *p* = .063). No significant differences were observed for POS' ethnicity *X* ^2^ (1, *N* = 111) = 1.4, *p* = .254, relationship status *X* ^2^ (1, *N* = 111) = 1.7, *p* = .678, or employment status *X* ^2^ (1, *N* = 111) = .003, *p* = .956. However, more POS in the OCD group were male *X* ^2^ (1, *N* = 105) = 7.5, *p* = .006. There was a significant difference in dyads living together more frequently in the OCD group, both historically (*X* ^2^ (1, *N* = 111) = 10.1, *p* = .001) and currently (*X* ^2^ (1, *N* = 111) = 4.3, *p* = .039).

The severity of functional impairment for PWC was not significantly different between OCD and HD groups, as assessed by comparing WSAS scores with an independent samples *t*‐test, *t*_(109)_ = .84, *p* = .404.

### Primary analysis

**FIGURE 1:**
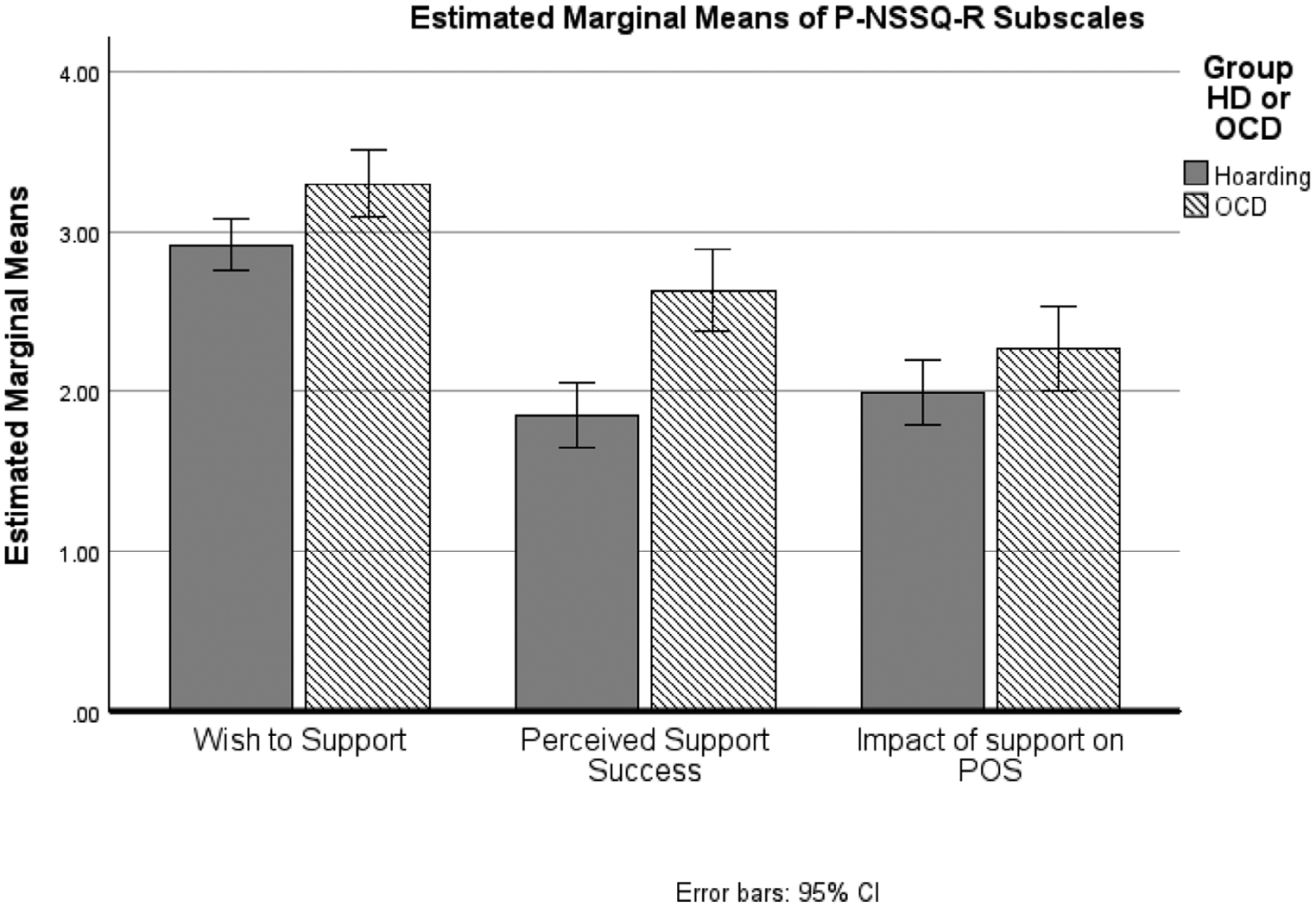
P‐NSSQ‐R subscale score by group.

The primary analysis used a 2 × 3 mixed model ANOVA with condition as the grouping variable and the within‐subject variable being subscales of the P‐NSSQ‐R: (wish to support; perceived support success; and impact of support on the POS). The main effect of subscale was significant, *F*_(2,218)_ = 77.0, *p* < .001, as was the main effect of group, *F*_(1,109)_ = 16.2, *p* < .001. These effects were modified by a significant interaction effect, *F*_(2,218)_ = 4.7, *p* = .01 (see Figure 1).

Multiple comparisons revealed that the POS(HD) reported both significantly lower wish to support (*t*_(109)_ = −2.9, *p* = .005) and lower perceived support success (*t*_(109)_ = −4.7, *p* < 001) relative to POS(OCD) (see Table 2). There was no difference between groups in rating the impact of support on the POS (*t*_(109)_ = −1.7, *p =* .098). POS also reported similar levels of burden as rated on the BSFC, *t*_(99)_ = .84, *p* = .465.

**TABLE 2:** Between‐groups scores on P‐NSSQ‐R subscales and BSFC (primary analysis) with multiple comparisons.

	Group				Between‐groups comparison	
	HD (*n* = 69)		OCD (*n* = 42)			
	*M*	*SD*	*M*	*SD*	*t*	*p*
1‐Wish to Support Subscale^b^	2.9	.74	3.3	.54	−.2.9	.005*
2‐Perceived support success^b^	1.9	.90	2.6	.74	−4.7	<.001*
3‐Impact of support on POS^b^	2.0	.87	2.3	.82	−1.7	.098
Reciprocity subscale^b^	1.9	1.1	2.6	1.0	−3.5	<.001*
Contact subscale^b^, ^c^	2.9	.80	3.4	.80	2098.5	<.001*
BSFC total score^d^	24.8	15.0	26.2	9.4	.84	.465

	Matched Pair	Group	*df*	*t*	*p*
Within groups comparison	1 vs. 2	HD (*n* = 69)	68	9.7	<.001*
		OCD (*n* = 42)	41	6.2	<.001*
	1 vs. 3	HD (*n* = 69)	68	8.7	<.001*
		OCD (*n* = 42)	41	6.7	<.001*
	2 vs. 3	HD (*n* = 69)	68	−1.5	.141
		OCD (*n* = 42)	41	2.3	.027*

^b^ P‐NSSQ‐R subscales divided by the number of items for comparison.

^c^ Contact subscale data not normally distributed in OCD group, Mann–Whitney test used.

^d^ BSFC completed by 101 POS, (HD group *n* = 59, OCD group *n* = 42).

*Significant at .05 level.

Paired‐Samples *t*‐tests revealed that within both groups, POS reported higher wish to support versus perceived support success (HD: *t*_(68)_ = 9.7, *p* < .001, OCD: *t*_(41)_ = 6.2, *p* < .001), and versus the impact of support on the POS (HD: *t*_(68)_ = 8.7, *p* < .001, OCD: *t*_(41)_ = 6.7, *p* < .001).

The P‐NSSQ‐R contact subscale data were skewed at the upper limit in the OCD group. A Mann–Whitney *U* test found that POS(OCD) reported a higher frequency of contact with the PWC compared with the HD group (*U* = 2098.5, *p* > .001). Follow‐up partitioned Chi‐square tests were used to investigate differences in individual items. All but one contact subscale item showed no significant between‐groups difference. For the frequency of face‐to‐face contact occurring in PWC homes, weekly in‐home contact was significantly less frequently reported in the HD group *X* ^2^ (1, *N* = 111) = 7.2, *p* = .007. An independent‐samples t‐test comparing total scores on the P‐NSSQ‐R reciprocity subscale showed a significant difference, with POS in the OCD group rating greater support available in turn from the PWC, *t*_(109)_ = −3.5, *p >* .001. Individual item mean scores for the P‐NSSQ‐R are provided in Data S8.

Overall, these results indicate that people supporting in the HD group rated both the wish to support and its perceived success as significantly lower than those supporting people with OCD. These groups did not differ in terms of the perceived impact of providing such support. The validity of this finding was aided by the groups not differing significantly on a measure of caregiver burden.

### Secondary analysis

#### Public stigma (CAMI)

**FIGURE 2:**
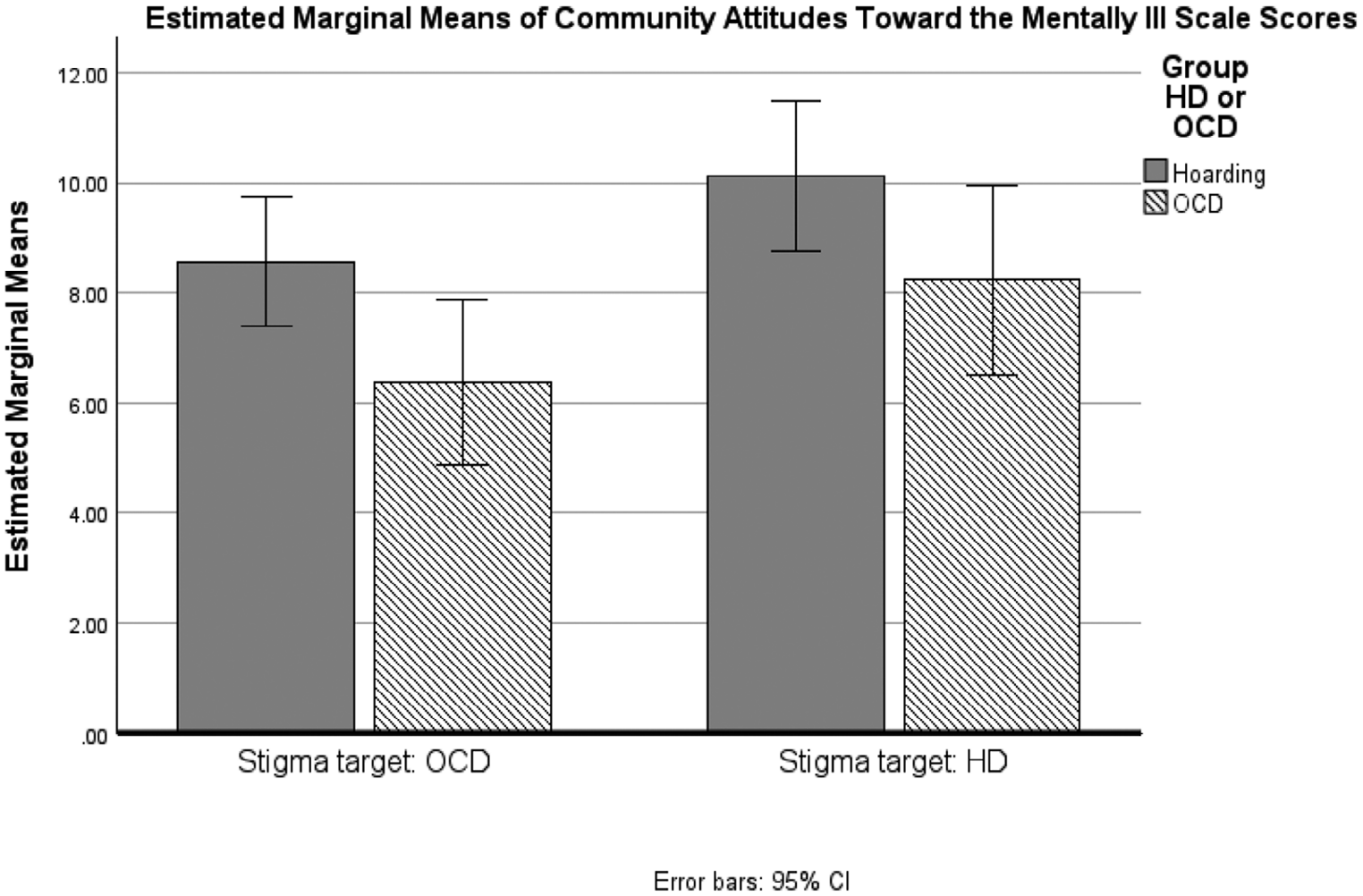
CAMI scores by group for public stigma attitudes towards Individuals with OCD versus HD.

The extent to which the POS separately stigmatized OCD and HD was analysed using a mixed model ANOVA. The main effect of the stigma target was significant, *F*_(1,107)_ = 15.9, *p* < .001, as was the main effect of group, *F*_(1,107)_ = 4.6, *p* = .034. The interaction was not significant, *F*_(1,107)_ = .13, *p* = .722. The group difference indicated that POS in the HD group reported higher perceived stigma overall for both diagnoses, with both groups rating HD more negatively (see Figure 2). Surprisingly, there was no evidence that being a supporter of somebody with either condition modified these ratings.

#### Associative stigma (ASS)

A mixed model ANOVA on the extent to which the POS feel themselves to be stigmatized by their association with the person (group x ASS subscales (affective, behavioural, cognitive)) found a significant main effect of subscale (*F*_(2,210)_ = 41.4, *p* < .001) and of the group (*F*_(1,105)_ = 9.6, *p* = .003). The interaction effect was not significant, *F*_(2,218)_ = 2.2, *p* = .111. Significantly greater levels of associative stigma were reported by POS(HD) (see Table 3).

**TABLE 3:** Between‐groups scores on additional self‐report measures (secondary analysis).

	Group						Between‐groups comparison	
	HD (*n* = 67)		OCD (*n* = 42)		Total (*n* = 109)			
	*M*	*SD*	*M*	*SD*	*M*	*SD*	*t*	*p*
CAMI								
Stigma target was OCD^e^	8.6	5.5	6.4	3.9	7.7	5.0	2.4	.016*
Stigma target was HD	10.1	5.8	8.2	5.3	9.4	5.7	1.7	.092

	HD (*n* = 65)		OCD (*n* = 42)		Total (*n* = 107)			
ASS								
ASS total score^e^	18.9	15.0	10.5	11.1	15.6	14.1	3.3	.001*
Affect^e^	7.6	5.5	4.4	4.4	6.3	5.3	3.4	<.001*
Behaviour^e^, ^f^	5.1	4.7	2.6	3.2	4.1	4.3	3.2	.002*
Cognition^e^	5.5	4.9	3.2	3.5	4.6	4.5	2.8	.006*

^e^ Adjusted for unequal variances.

^f^ Behaviour ASS subscale transformed for comparison.

*Significant at .05 level.

### Exploratory analysis

Due to a comorbidity rate of 33.3% in the total sample, the primary and secondary analyses were re‐run, omitting cases where POS identified the PWC as having comorbid OCD or HD (filtered group sizes HD *n* = 50, OCD *n* = 24, see Data S9). Similar main and interaction effects were found as per the whole sample grouping, other than that the main effect of the group in the public stigma model was no longer significant *F*_(1,70)_ = 3.6, *p* = .062, likely due to the decreased power. This analysis indicates that comorbidity was unlikely to have overestimated or underestimated any differences in the primary analysis.

A hierarchical regression model investigated the relationships between group and associative stigma (total ASS score) on the dependent variables of subscales of the P‐NSSQ‐R when controlling first for PWC age. The model for wish to support was significant (*F*_(3,106)_ = 4.6, *p* = .005) with age accounting for 4.2% of the variance (*R* ^2^ change = .042, *p* = .033), associative stigma an additional 5.0% (*R* ^2^ change = .33, *p* = .018) and group an additional 2.5% (*R* ^2^ change = .025, *p* = .091). The model for perceived support success was significant (*F*_(3,106)_ = 11.8, *p* < .001) with age accounting for 13.5% of the variance (*R* ^2^ change = .135, *p >* .001), associative stigma an additional 6.8% (*R* ^2^ change = .068, *p =* .003) and group an additional 5.3% (*R* ^2^ change = .053, *p* = .008). The model for the impact of support on the POS was significant (*F*_(3,106)_ = 19.0, *p* < .001) with age accounting for 2.3% of the variance (*R* ^2^ change = .023, *p* = .12), associative stigma an additional 33.0% (*R* ^2^ change = .33, *p* < .001) and group an additional .40% (*R* ^2^ change = .004, *p* = .45).

## DISCUSSION

Based on previous findings that people with HD rated their social support as less adequate than OCD controls, this study used the perspectives of those providing such support to evaluate the two factors which could explain that finding. Specifically, how much the POS *wishes* to offer support, and how *successful* they perceive that support to be. Both factors were expected to be associated with the degree of satisfaction in providing support. Results suggest that both factors differentiate between diagnoses. Those supporting people with HD reported a lower wish to support relative to POS in the OCD group, suggesting that support is being offered less willingly. Those supporting people with HD also viewed their support as less successful relative to OCD group counterparts, consistent with findings of lower ratings of ‘being supported’ by people with HD (Barton et al., 2021; Edwards et al., 2023). Interestingly, the hypothesis that POS(HD) would also report a more negative impact on them from offering support was not supported, with support experience not differing on the P‐NSSQ‐R subscale nor as measured more globally via burden. Note that samples were comparable in terms of levels of functional impairment due to their condition. The differences found in associative stigma and its relationship to wish to support and its success suggest that this could be a possible mechanism.

Previous literature finding poorer perceived support for individuals with HD (Barton et al., 2021; Edwards et al., 2023) can now be understood, at least in part, in relation to shortcomings in available support availability and provision. Another relevant factor was PWC age, which was associated with lower perceived success of support. Previous research has found associations between age and hoarding severity, noting uncertainty as to whether there are indeed differentiating age‐related factors or if cumulative accumulation of clutter over time is the primary cause (Drury et al., 2014). There is no comparative evidence to suggest that offering support to someone with HD would be a more difficult or burdensome experience than offering support to an individual with OCD, but this was tentatively considered in the context of HD carer research focusing on social isolation and carer frustration (Sampson, 2013; Wilbram et al., 2008). The findings did not indicate that these POS(HD) are at increased risk of withdrawing support due to carer burden, relative to OCD. We can tentatively consider that estrangement in HD more likely occurs through POS choosing to increase distance from the PWC, rather than reaching a point of overwhelm whilst maintaining their best efforts to continue to help.

Previous research has suggested how an inability to visit a person's home might lead to ambiguous loss of that relationship and associated gradual dwindling of support efforts (Sampson, 2013). Alternatively, it might be the case that the negative effects of accommodating hoarding symptoms are more salient to these POS and there is less motivation to add fuel to the fire through perceived collusion or unwanted actions, such as covert house clearances (Büscher et al., 2014). Where with OCD, there might be an idea that POS accommodating symptoms is ‘better than doing nothing’ (Kobori et al., 2017), could it be that HD POS begin to believe that reducing support is best? This aligns with Mayes et al. (2024) finding that with increased clutter, relatives experienced less benefit from offering support and less certainty that the PWC were getting the right help. It would also appear that PWC' age plays a role in POS' perception of support success; possibly the longer that clutter remains the less hopeful and involved a supporter might become.

Those supporting PWC(HD) in this sample were also found to experience more associative stigma (supporters' own sense of stigma due to their connection to the stigmatized individual), and to have more negative attitudes towards both OCD and HD, relative to POS(OCD). Note that POS in both the HD and OCD groups rated HD as a more stigmatized condition on a measure of public stigma, as found in a previous comparison of HD and OCD public stigma (Chasson et al., 2018). Previous research found that proximity reduced a sense of difference in individuals with HD but not disdain or blame towards them (Chasson et al., 2018). Research has already established that relatives of a person with HD may become socially isolated themselves (Sampson, 2013; Wilbram et al., 2008). The present findings suggest that associative stigma from internalizing persecutory ideas about mental health conditions is significantly elevated in POS to those with HD. This highlights the need for larger‐scale trialling of interventions involving these supporters, beyond the successful proof‐of‐concept studies completed thus far (Chasson et al., 2014; Thompson et al., 2016), to help these people to manage associative stigma and develop a clearer understanding of how they can support the PWC to achieve more positive therapeutic outcomes.

### Limitations

The findings of this study have been used to infer what might be happening between POS–PWC dyads in the context of HD. Having established a deficit in support quality, further research involving dyads of supporters and support is needed to investigate causal factors and clinical interventions which increase support quality. It is also recognized that the primary analysis was based on the adaptation of the NSSQ‐R beyond its originally intended use to the point of developing a novel scale. Whilst this poses a challenge to the validity of the findings, the scale was adapted with reference to existing literature and what PWC, from clinical experience, report to be key aspects of offering support and measuring support quality in the absence of a readily available alternative measure. Nonetheless, future research involving dyads could aid construct validity by testing the P‐NSSQ‐R's correlation with existing validated measures for related concepts, such as familial rejection (Tolin et al., 2008).

It is possible that recruiting supporters who self‐identified in the context of hoarding may have validity issues; the use of the Clutter Image Rating as a secondary selection criterion goes some way towards addressing this issue.

Dyads more commonly lived together in the OCD group and it could be that lower associative stigma and enhanced support were attributable to proximity. Higher rates of PWC having a diagnosis, having sought professional help, and having more frequent contact in the PWC' home were expected for the OCD group, in line with previous literature describing barriers in these factors for individuals with HD. However, future research could seek to recruit matched samples to increase confidence in the specificity of findings.

This study recruited via social media and advertising through relevant charities which may have introduced a sampling bias. It is unlikely that this recruitment method would have appealed to POS who were actively choosing not to engage in support activities, or who were estranged from the PWC that they had a connection to, supported by the high frequency of ongoing contact with the PWC reported in this sample. Future research could seek a broader range of engagement in current support activity. Also related to recruitment is the high level of comorbidity, although this was mitigated by repeating the main analysis with smaller but more specific groups.

### Study implications

Across both OCD and HD groups, these POS reported a higher wish to support than the perceived level of success they believe to be achieving. This demonstrates the potential usefulness of involving partners and family members in psychological therapy and POS learning alternative strategies to accommodating symptoms. Whilst this is becoming better recognized in OCD treatment, family‐integrated CBT for HD is lagging behind. Involving POS may hold specific value for HD treatment, addressing hopelessness before POS are no longer prepared to offer help. Clinicians should also consider that with HD, POS may have a particular vulnerability to associative stigma and help POS acknowledge and resist stigma.

Further research should involve POS and PWC to understand *why* support for HD seems to be relatively less well delivered and received. Recruiting dyads would add reliability to future studies and provide a means of experimentally investigating interpersonal factors, such as warmth, trust, and stigma‐related concepts such as disdain (Chasson et al., 2018). Longitudinal research could chart the provision and quality of support for PWC with HD over time, helping to identify factors associated with estrangement and with the maintenance of long‐term supportive relationships.

## AUTHOR CONTRIBUTIONS

**James Dennis:** Conceptualization; investigation; writing – original draft; methodology; validation; writing – review and editing; formal analysis; project administration; visualization; software; data curation. **Kate Rosen:** Writing – review and editing; supervision; resources. **Paul Salkovskis:** Conceptualization; methodology; writing – review and editing; supervision; resources.

## CONFLICT OF INTEREST STATEMENT

Paul Salkovskis is a member of the editorial board of the British Journal of Clinical Psychology, there has been no involvement in the editorial process for this submission. James Dennis and Kate Rosen declare no conflicts of interest.

## Supporting information


Appendix S1.

## Data Availability

Data are not publicly available in accordance with conditions set by local ethical body approval.
