# Supplementary material for: ‘Not my mess’?: How do supporters of individuals with hoarding difficulties rate the quality of the support they offer?
Source: Br J Clin Psychol. 2024 Nov 22;64(2):475–90. doi: 10.1111/bjc.12520 (PMC12057303; doi:10.1111/bjc.12520)
Supplement: Supplementary file 1 — Appendix S1. [file BJC-64-475-s001.docx]

## Supplementary Information

A: Study recruitment poster

B: Study information sheet

C: Ethical approval

D: Paper Survey Form

E: P-NSSQ-R Items and Scoring Guide

F: P-NSSQ-R pilot and reliability analysis

G: Full demographics table

H: Adapted Norbeck Full Scores

I: Full description of supplementary analysis with grouping excluding comorbid OCD and HD

## Supplementary Information A: Study recruitment poster


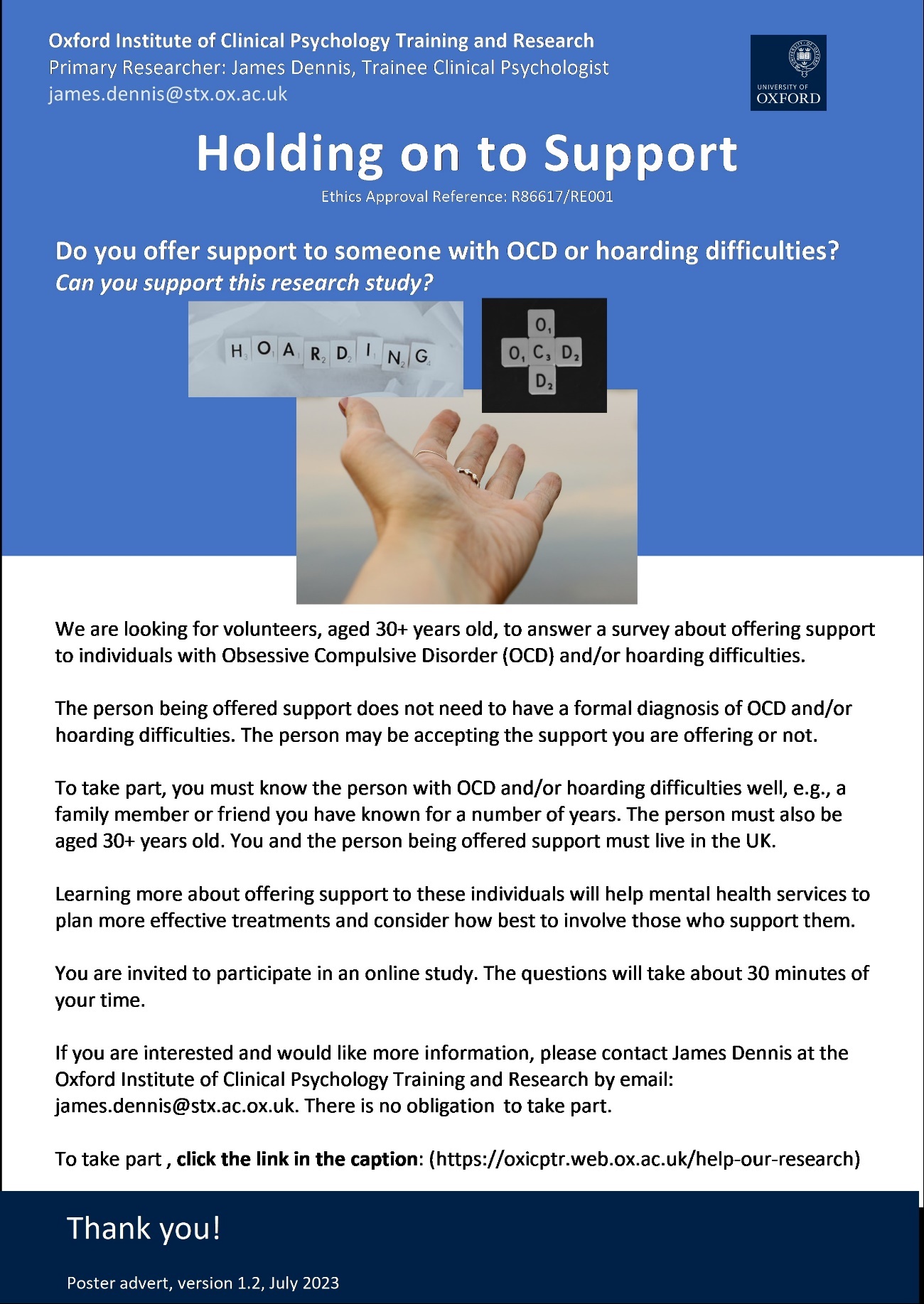


## Supplementary Information B: Study information sheet

| **Oxford Institute of Clinical Psychology Training and Research**  Principal Investigator (Project supervisor): Paul Salkovskis, Professor of Clinical Psychology, Department of Experimental Psychology, University of Oxford [paul.salkovskis@hmc.ox.ac.uk](mailto:paul.salkovskis@hmc.ox.ac.uk)  Primary Researcher: James Dennis, Trainee Clinical Psychologist [james.dennis@stx.ox.ac.uk](mailto:james.dennis@stx.ox.ac.uk) | 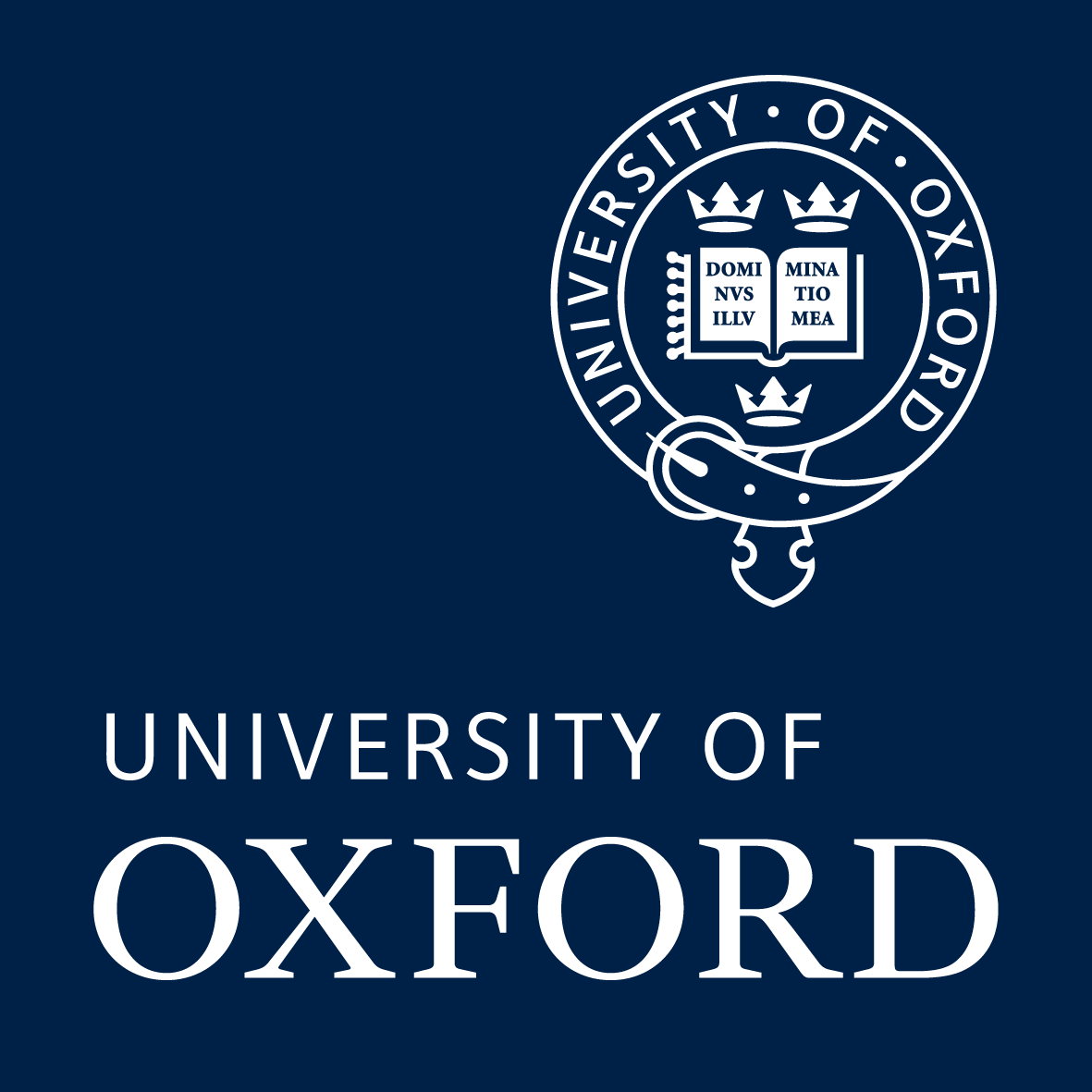 |
| --- | --- |

**Holding on to Support**

**CUREC Approval Reference:** R86617/RE001

**General Information**

Hoarding difficulties and Obsessive Compulsive Disorder (OCD) affect many people and can cause upset and danger for the person. Research suggests that people with these problems are also at risk of becoming disconnected from their friends and family.

This study will survey people who offer support to people with hoarding difficulties and OCD. The questions will ask about the help and support they give, or try to give. This will help us to understand any differences in how people with hoarding difficulties and OCD are offered support. Knowing this will help plan more effective treatments and how best to involve those who support them.

We appreciate your interest in participating in this questionnaire. You have been invited to participate as you are 30 years of age or above and you offer support to somebody with hoarding difficulties and/or OCD that you have a personal connection to (e.g., a family member or friend you have known for a number of years?). The person with hoarding difficulties and/or OCD should also be 30 years of age or above.

*Please read through this information before agreeing to participate (if you wish to) by ticking the ‘yes’ box below.*

You may ask any questions before deciding to take part by contacting the researcher (details below).

The Primary Researcher is James Dennis (Trainee Clinical Psychologist, The Oxford Institute of Clinical Psychology Training and Research). This project is being completed under the supervision of Prof Paul Salkovskis (Professor of Clinical Psychology, Department of Experimental Psychology), and Dr Kate Rosen (Consultant Clinical Psychologist, Oxford Cognitive Therapy Centre and OHSPIC).

You will be asked to respond to a number of questions, most of which have multiple options. It is important that you try to answer all of the questions. This should take about 30 minutes. No specialist background knowledge is required.

**Do I have to take part?**

No. Please note that participation is voluntary. If you do decide to take part, you may withdraw at any point for any reason before submitting your answers by pressing the ‘Exit’ button/ closing the browser.

**How will my data be used?**

We will not collect any data that could directly identify you. Your IP address will not be stored.

The responses you provide will be stored in a password-protected electronic file on University of Oxford secure servers and may be used in academic publications, conference presentations, reports for external organisations, websites, videos. Research data will be stored for three years after publication or public release of the work of the research.

The results will be written up for a DClin Psych degree.

**Who has reviewed this study?**

This project has been reviewed by, and received ethics clearance through, a subcommittee of the University of Oxford Central University Research Ethics Committee [R86617/RE001].

**Who do I contact if I have a concern or I wish to complain?**

If you have a concern about any aspect of this study, please speak to James Dennis ([james.dennis@stx.ox.ac.uk](mailto:james.dennis@stx.ox.ac.uk)) or their supervisor Paul Salkovskis, and we will do our best to answer your query. We will acknowledge your concern within 10 working days and give you an indication of how it will be dealt with. If you remain unhappy or wish to make a formal complaint, please contact the Chair of the Research Ethics Committee at the University of Oxford who will seek to resolve the matter as soon as possible:

Medical Sciences Interdivisional Research Ethics Committee; Email: ethics@medsci.ox.ac.uk; Address: Research Services, University of Oxford, Boundary Brook House, Churchill Drive, Headington, Oxford OX3 7GB

Please note that you may only participate in this survey if:

- You are 30 years of age or over.
- You live in the UK.
- You offer support to somebody with hoarding difficulties and/or OCD that you have a personal connection to, e.g., a family member or friend you have known for a number of years.
- The person you offer support to is 30 years of age or over.
- The person you offer support to lives in the UK.
- You agree: to take part in this study; to your responses being included in a dataset that shall be analysed and written up as part of a doctoral thesis; and academic journal publication.

If you have read the information above and agree to participate with the understanding that the data (including any personal data) you submit will be processed accordingly, please click the link to start. <link>

## Supplementary Information C: Ethical approval


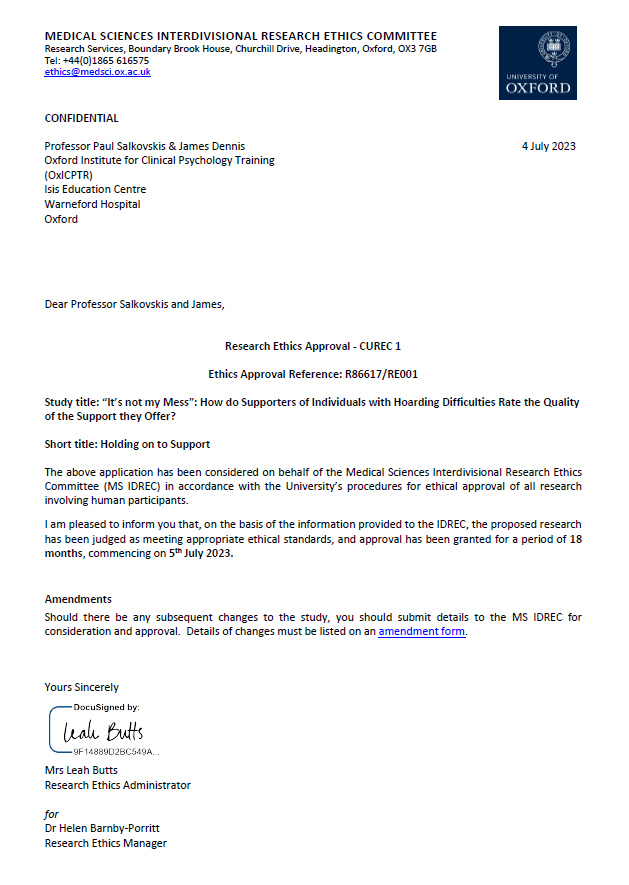


## Supplementary Information D: Paper Survey Form

*Please note: The paper form was not requested for use by any respondents. The items are equivalent to those included in the online Qualtrics survey.*


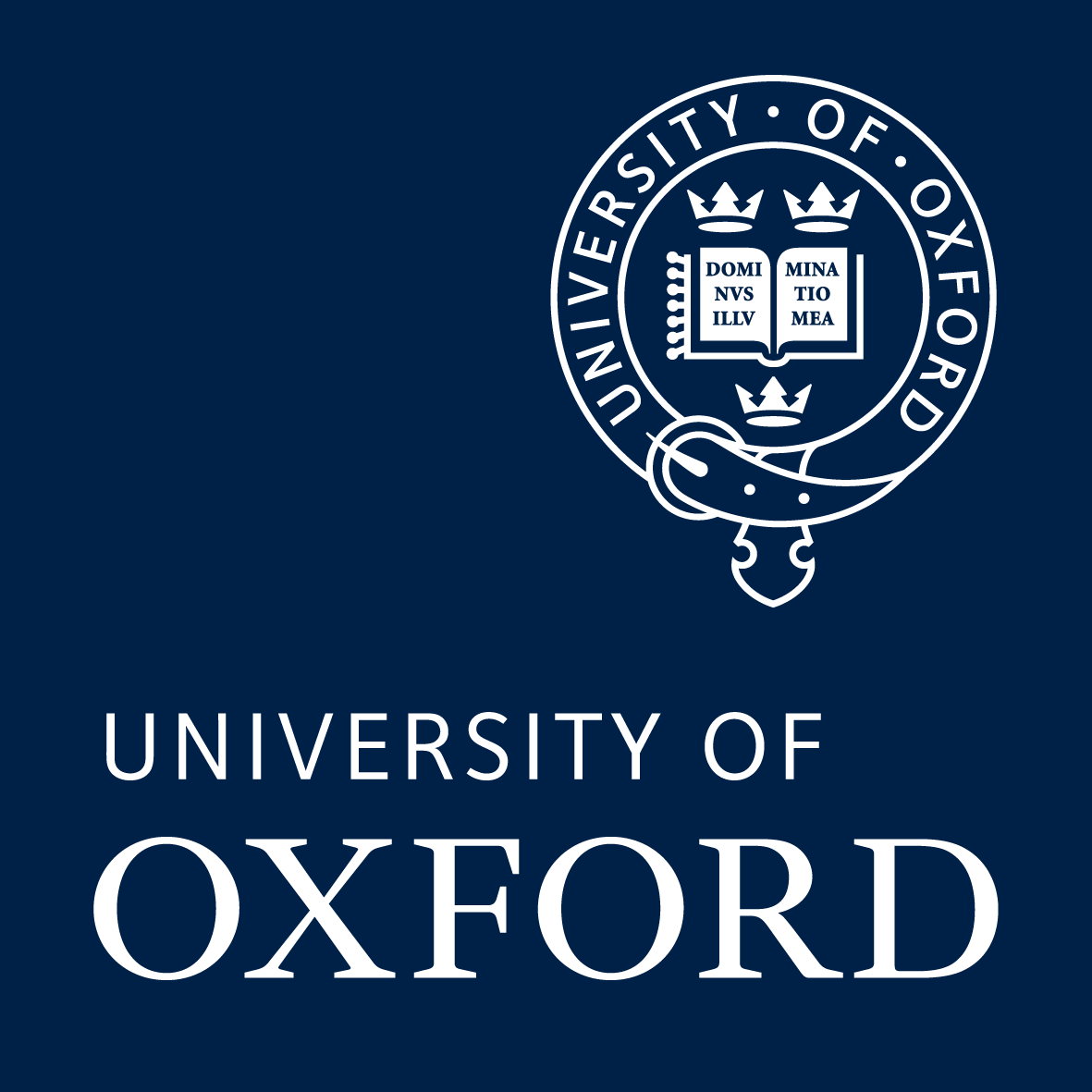
**Oxford Institute of Clinical Psychology Training and Research**

Principal Investigator (Project supervisor): Paul Salkovskis, Professor of Clinical Psychology, Department of Experimental Psychology, University of Oxford

Primary Researcher: James Dennis, Trainee Clinical Psychologist [james.dennis@stx.ox.ac.uk](mailto:james.dennis@stx.ox.ac.uk)

**Holding on to Support**

**CUREC Approval Reference:** R86617/RE001

**Please note that you may only participate in this survey if:**

- You are 30 years of age or over.
- You live in the UK.
- You offer support to somebody with hoarding difficulties and/or OCD that you have a personal connection to, e.g., a family member or friend you have known for a number of years.
- The person you offer support to is 30 years of age or over.
- The person you offer support to lives in the UK.
- You agree: to take part in this study; to your responses being included in a dataset that shall be analysed and written up as part of a doctoral thesis; and academic journal publication.

**If you have read the information above and agree to participate with the understanding that the data (including any personal data) you submit will be processed accordingly, please tick the box below to start.**

□Yes, I have read the criteria and information sheet and I wish to take part

□No, I do not wish to take part

**Demographics**

The following questions relate to **the person that you offer support to**

- Can you confirm that the person lives in the UK?
  - Yes
- Obsessive compulsive disorder (OCD) is a mental health condition where a person has obsessive thoughts and compulsive behaviours.

Obsessive compulsive disorder (OCD) affects people differently, but usually causes a particular pattern of thoughts and behaviours.

An obsession is an unwanted and unpleasant thought, image or urge that repeatedly enters your mind, causing feelings of anxiety, disgust or unease. A compulsion is a repetitive behaviour or mental act that you feel you need to do to temporarily relieve the unpleasant feelings brought on by the obsessive thought.

NHS (2023) *Obsessive compulsive disorder (OCD)* (<https://www.nhs.uk/mental-health/conditions/obsessive-compulsive-disorder-ocd/>)

Does the person you offer support to have OCD?

- - Yes
  - No
- A hoarding disorder is where someone acquires an excessive number of items and stores them in a chaotic manner, usually resulting in unmanageable amounts of clutter. The items can be of little or no monetary value.

Hoarding is considered a significant problem if: the amount of clutter interferes with everyday living – for example, the person is unable to use their kitchen or bathroom and cannot access rooms the clutter is causing significant distress or negatively affecting the quality of life of the person or their family – for example, they become upset if someone tries to clear the clutter and their relationship suffers

NHS (2022) *Hoarding Disorder* (<https://www.nhs.uk/mental-health/conditions/hoarding-disorder/>)

Does the person you offer support to have hoarding disorder?

- - Yes
  - No
- If the person has both OCD and hoarding disorder, which condition would you consider to be their main ‘problem’?
  - Hoarding Disorder
  - Obsessive Compulsive Disorder
- As far as you can tell, does the person you offer support to consider *themselves* to have any of these conditions?
  - Hoarding Disorder
  - Obsessive Compulsive Disorder
  - Does not consider themselves to have either condition
- If the person you offer support to considers themselves to have both OCD and hoarding disorder, which condition do *they* consider to be their main ‘problem’?
  - Hoarding Disorder
  - Obsessive Compulsive Disorder
- Does the person have a formal diagnosis?
  - Hoarding Disorder
  - Obsessive Compulsive Disorder
  - Hoarding Disorder and OCD
  - No formal diagnosis of Hoarding Disorder nor Obsessive Compulsive Disorder
- How many years ago did the person first have symptoms of OCD and/or hoarding difficulties?
  - …………
- To the best of your knowledge, has the person ever sought help for their condition?
  - Yes
  - No
- What is their age? (in years)
  - …………

- What is their gender identity?
  - Woman
  - Man
  - Non-binary
  - Transgender
  - Other
  - Prefer not to say
- What is their ethnicity?

White:

- - English / Welsh / Scottish / Northern Irish / British
  - Irish
  - Gypsy or Irish Traveller
  - Any other White Background

Mixed / Multiple Ethnic Groups:

- - White and Black Caribbean
  - White and Black African
  - White and Asian
  - Any other Mixed / Multiple Ethnic Background

Asian / Asian British:

- - Indian
  - Pakistani
  - Bangladeshi
  - Chinese
  - Any other Asian Background

Black / African / Caribbean / Black British:

- - African
  - Caribbean
  - Any other Black / African / Caribbean Background

Other Ethnic Group:

- - Arab
  - Any other ethnic Background:
- What is the person’s marital status?
  - Single
  - Married or in a civil partnership
  - Cohabiting
  - Divorced or separated
  - Widowed
- What is the person’s employment status?
  - Employed full-time
  - Employed part-time
  - Unemployed
  - Retired
  - Volunteering
- What is their highest level of formal education?
  - No formal Qualification
  - GCSEs/GNVQs/Scottish Standard
  - A-Levels/BTEC/Scottish Higher
  - Apprenticeship
  - Graduate Degree (e.g. BA, BSc)
  - Higher Degree (e.g. MA, MSc, PGCE)
  - Doctorate (e.g. PhD, EdD)
  - Prefer not to say

The following questions are about **you**

- Can you confirm that you live in the UK?
  - Yes

- What is your age? (in years)
  - …………

- What is your gender identity?
  - Woman
  - Man
  - Non-binary
  - Transgender
  - Other
  - Prefer not to say
- What is your ethnicity?

White:

- - English / Welsh / Scottish / Northern Irish / British
  - Irish
  - Gypsy or Irish Traveller
  - Any other White Background

Mixed / Multiple Ethnic Groups:

- - White and Black Caribbean
  - White and Black African
  - White and Asian
  - Any other Mixed / Multiple Ethnic Background

Asian / Asian British:

- - Indian
  - Pakistani
  - Bangladeshi
  - Chinese
  - Any other Asian Background

Black / African / Caribbean / Black British:

- - African
  - Caribbean
  - Any other Black / African / Caribbean Background

Other Ethnic Group:

- - Arab
  - Any other ethnic Background:
- What is your marital status?
  - Single
  - Married or in a civil partnership
  - Cohabiting
  - Divorced or separated
  - Widowed
- What is your employment status?
  - Employed full-time
  - Employed part-time
  - Unemployed
  - Retired
  - Volunteering
- What is your highest level of formal education?
  - No formal Qualification
  - GCSEs/GNVQs/Scottish Standard
  - A-Levels/BTEC/Scottish Higher
  - Apprenticeship
  - Graduate Degree (e.g. BA, BSc)
  - Higher Degree (e.g. MA, MSc, PGCE)
  - Doctorate (e.g. PhD, EdD)
  - Prefer not to say
- What is your relationship to the person you offer support?
  - Spouse or partner
  - Significant other
  - Family member or relative
  - Neighbour
  - Friend
  - Work associate
  - Health care provider
  - Counsellor or therapist
  - Minister/Priest/Rabbi/Imam
  - Other
- If ‘other’, please specify your relationship with the person you offer support.
  - ……………………………………….

- Have you, or are you currently, living with the person with OCD and/or hoarding difficulties?
  - Currently, for more than 10 years
  - Currently, for less than 10 years
  - Previously, for more than 10 years
  - Previously, for less than 10 years
  - Never lived together

**Measures**

**Adapted Work and Social Adjustment Scale**

People's problems sometimes affect their ability to do certain day-to-day tasks in their lives.

To rate the impact of the person’s problem (hoarding difficulties OR OCD) look at each section and determine on the scale provided how much the problem impairs their ability to carry out the activity.

Please rate the responses on behalf of the person that you offer support to.

1. Because of their problem their ability to work is impaired. ‘0’ means ‘not at all impaired’ and ‘8’ means very severely impaired to the point they can't work. If the person is retired or chooses not to have a job for reasons unrelated to their problem, do not select an option below, and tick here: □

| 0 | 1 | 2 | 3 | 4 | 5 | 6 | 7 | 8 |
| --- | --- | --- | --- | --- | --- | --- | --- | --- |
| Not at all |  | Slightly |  | Definitely |  | Markedly |  | Very severely |

1. Because of their problem their home management (cleaning, tidying, shopping, cooking, looking after home or children, paying bills) is impaired.

| 0 | 1 | 2 | 3 | 4 | 5 | 6 | 7 | 8 |
| --- | --- | --- | --- | --- | --- | --- | --- | --- |
| Not at all |  | Slightly |  | Definitely |  | Markedly |  | Very severely |

1. Because of their problem their social leisure activities (with other people e.g. parties, bars, clubs, outings, visits, dating, home entertaining) are impaired.

| 0 | 1 | 2 | 3 | 4 | 5 | 6 | 7 | 8 |
| --- | --- | --- | --- | --- | --- | --- | --- | --- |
| Not at all |  | Slightly |  | Definitely |  | Markedly |  | Very severely |

1. Because of their problem, their private leisure activities (done alone, such as reading, gardening, collecting, sewing, walking alone) are impaired.

| 0 | 1 | 2 | 3 | 4 | 5 | 6 | 7 | 8 |
| --- | --- | --- | --- | --- | --- | --- | --- | --- |
| Not at all |  | Slightly |  | Definitely |  | Markedly |  | Very severely |

1. Because of their problem, their ability to form and maintain close relationships with others, including those they live with, is impaired.

| 0 | 1 | 2 | 3 | 4 | 5 | 6 | 7 | 8 |
| --- | --- | --- | --- | --- | --- | --- | --- | --- |
| Not at all |  | Slightly |  | Definitely |  | Markedly |  | Very severely |

**Clutter Images Rating Scale**

Kitchen

Please select the photo below that most accurately reflects the amount of clutter in their room


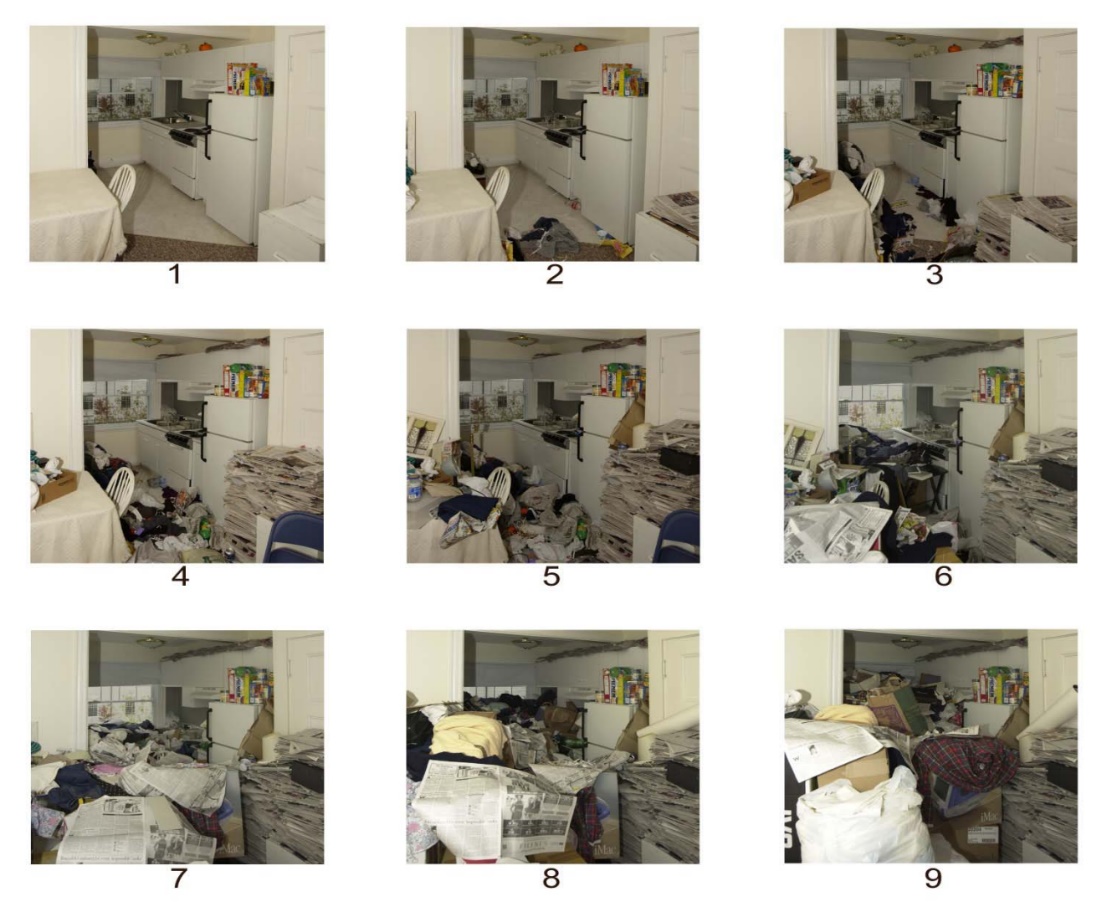


Bedroom

Please select the photo below that most accurately reflects the amount of clutter in their room


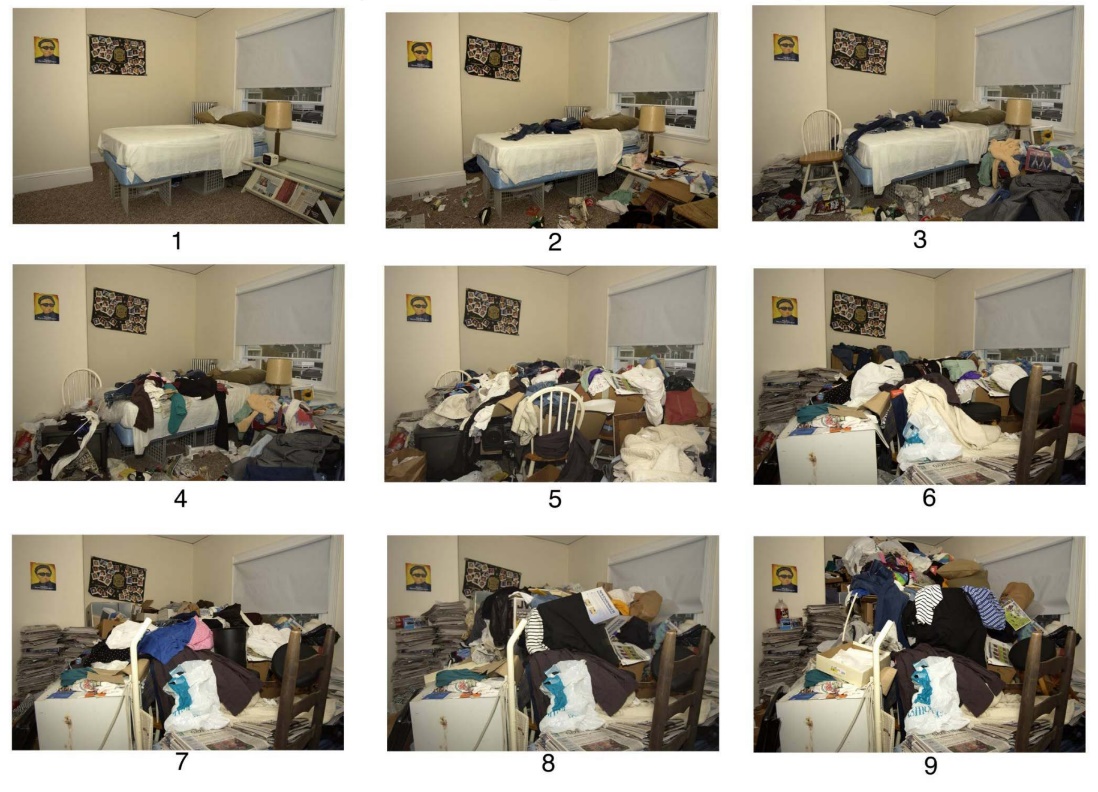


Living room

Please select the photo below that most accurately reflects the amount of clutter in their room


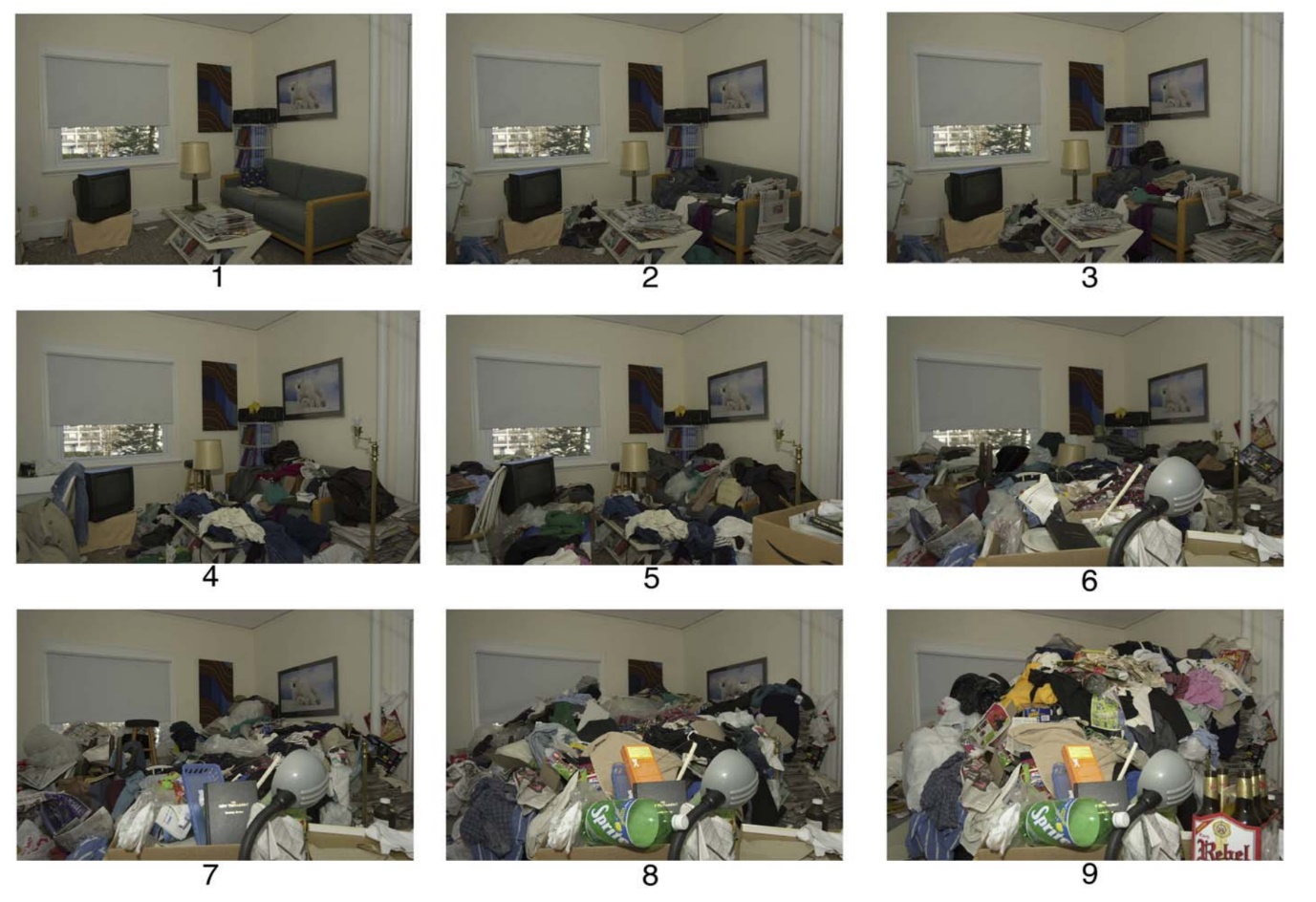


**Proxy Revised Norbeck Social Support Questionnaire**

- In years, how long have you known this person?
  - …………
- How frequently do you usually attempt to make contact with this person via phone call/letter/email/social media?
  - Daily
  - Weekly
  - Monthly
  - A few times a year
  - Once a year or less
- How often does the person usually respond?
  - Every time
  - Most of the time
  - Half the time or more
  - Less than half the time
  - Rarely or never
- How frequently does the person usually attempt to make contact with you via phone call/letter/email/social media?
  - Daily
  - Weekly
  - Monthly
  - A few times a year
  - Once a year or less
- How often do you usually respond?
  - Every time
  - Most of the time
  - Half the time or more
  - Less than half the time
  - Rarely or never
- How frequently do you usually see this person face-to-face at your home or in a public place?
  - Daily
  - Weekly
  - Monthly
  - A few times a year
  - Once a year or less
- How frequently do you usually see this person in their home?
  - Daily
  - Weekly
  - Monthly
  - A few times a year
  - Once a year or less
- When was your most recent contact with this person?
  - Yesterday
  - Last week
  - Last month
  - In the last year
  - More than a year ago

|  | | **Not at all** | | **A little** | **Moderately** | **Quite a bit** | **A great deal** |
| --- | --- | --- | --- | --- | --- | --- | --- |
| How much do you try to make the person feel liked or loved? | |  | |  |  |  |  |
| How much do you actually make this person feel liked or loved? | |  | |  |  |  |  |
| How much do you try to make this person feel respected or admired? | |  | |  |  |  |  |
| How much do you actually make this person feel respected or admired? | |  | |  |  |  |  |
| How open are you to this person confiding in you? | |  | |  |  |  |  |
| How much does the person confide in you? | |  | |  |  |  |  |
| How much do you agree with or support this person’s actions or thoughts regarding over acquiring and keeping possessions and/or obsessional and compulsive problems? | |  | |  |  |  |  |
| How much do you agree with or support this person’s actions or thoughts (other than matters regarding over acquiring and keeping possessions and/or obsessional and compulsive problems)? | |  | |  |  |  |  |
|  | **Not at all** | | **A little** | | **Moderately** | **Quite a bit** | **A great deal** |
| How available are you to help and support the person to do what they need to do? |  | |  | |  |  |  |
| How much does the person accept help and support from you with doing what they need to do? |  | |  | |  |  |  |
| How available are you to help and support the person meet their basic needs? |  | |  | |  |  |  |
| How much does the person accept help and support from you with meeting their basic needs? |  | |  | |  |  |  |
| If the person needed help and support for several weeks, how available could you be to help this person? |  | |  | |  |  |  |
| How much would the person accept help and support from you if you offered this for several weeks? |  | |  | |  |  |  |
| If the person needed help and support in an emergency, how available could you usually be? |  | |  | |  |  |  |
| How much would the person accept help and support from you in an emergency? |  | |  | |  |  |  |

|  | **Not at all** | **A little** | **Moderately** | **Quite a bit** | **A great deal** |
| --- | --- | --- | --- | --- | --- |
| How warmly does the person respond to your efforts to help and support them? |  |  |  |  |  |
| Is the person grateful of your efforts to help and support them? |  |  |  |  |  |
| Does the person recognise the effort you put into helping and supporting them? |  |  |  |  |  |
| Is the person welcoming of your help and support? |  |  |  |  |  |
| Does the person reject your help and support? |  |  |  |  |  |
| How pointless does it feel to offer help and support to the person? |  |  |  |  |  |
| How confident are you that you are offering the right kind of help and support to the person? |  |  |  |  |  |
| Does the help and support you offer to the person make a difference? |  |  |  |  |  |

|  | **Not at all** | **A little** | **Moderately** | **Quite a bit** | **A great deal** |
| --- | --- | --- | --- | --- | --- |
| How frustrated do you feel when offering help and support to the person? |  |  |  |  |  |
| How often do you snap at the person when offering help and support? |  |  |  |  |  |
| How satisfied are you that you do your best to help and support to the person? |  |  |  |  |  |
| Do you feel hopeless about offering help and support to the person? |  |  |  |  |  |
| Do you get angry with the person when offering help and support? |  |  |  |  |  |
| Do you find helping and supporting the person rewarding? |  |  |  |  |  |
| Do you feel resentful towards the person that you help and support? |  |  |  |  |  |
| Do you feel guilty about not offering enough help and support to the person? |  |  |  |  |  |

|  | **Not at all** | **A little** | **Moderately** | **Quite a bit** | **A great deal** |
| --- | --- | --- | --- | --- | --- |
| How much does this person make you feel liked or loved? |  |  |  |  |  |
| How much does this person make you feel respected or admired? |  |  |  |  |  |
| How much can you confide in this person? |  |  |  |  |  |
| How much does this person agree with or support your actions or thoughts (other than matters regarding over acquiring and keeping possessions and/or obsessional and compulsive problems)? |  |  |  |  |  |
| If you needed immediate help, how much could this person usually help? |  |  |  |  |  |
| If you needed help for several weeks, how much could this person help you? |  |  |  |  |  |

**Community Attitudes Toward the Mentally Ill Scale**

The following statements express various opinions about **Obsessive Compulsive Disorder** and the people who have this condition.

Please indicate the response which most accurately describes your reaction to each statement. It's your first reaction which is important. Don't be concerned if some statements seem similar to ones you have previously answered. Please be sure to answer all statements.

|  | **Agree Strongly** | **Agree Slightly** | **Neither Agree nor disagree** | **Disagree Slightly** | **Disagree Strongly** |
| --- | --- | --- | --- | --- | --- |
| One of the main causes of OCD is a lack of self- discipline and will-power |  |  |  |  |  |
| Virtually anyone can develop OCD |  |  |  |  |  |
| People with OCD have for too long been the subject of ridicule |  |  |  |  |  |
| We need to adopt a far more tolerant attitude toward people with OCD in our society |  |  |  |  |  |
| We have a responsibility to provide the best possible care for people with OCD |  |  |  |  |  |
| People with OCD don't deserve our sympathy |  |  |  |  |  |
| There are sufficient existing services for people with OCD |  |  |  |  |  |
| I would not want to live next door to someone who has OCD |  |  |  |  |  |
| No-one has the right to exclude people with OCD from their neighbourhood |  |  |  |  |  |
| The best therapy for many people with OCD is to be part of a normal community |  |  |  |  |  |

**Community Attitudes Toward the Mentally Ill Scale**

The following statements express various opinions about **hoarding difficulties** and the people who have this condition.

Please indicate the response which most accurately describes your reaction to each statement. It's your first reaction which is important. Don't be concerned if some statements seem similar to ones you have previously answered. Please be sure to answer all statements.

|  | **Agree Strongly** | **Agree Slightly** | **Neither Agree nor disagree** | **Disagree Slightly** | **Disagree Strongly** |
| --- | --- | --- | --- | --- | --- |
| One of the main causes of hoarding difficulties is a lack of self- discipline and will-power |  |  |  |  |  |
| Virtually anyone can develop hoarding difficulties |  |  |  |  |  |
| People with hoarding difficulties have for too long been the subject of ridicule |  |  |  |  |  |
| We need to adopt a far more tolerant attitude toward people with hoarding difficulties in our society |  |  |  |  |  |
| We have a responsibility to provide the best possible care for people with hoarding difficulties |  |  |  |  |  |
| People with hoarding difficulties don't deserve our sympathy |  |  |  |  |  |
| There are sufficient existing services for people with hoarding difficulties |  |  |  |  |  |
| I would not want to live next door to someone who has hoarding difficulties |  |  |  |  |  |
| No-one has the right to exclude people with hoarding difficulties from their neighbourhood |  |  |  |  |  |
| The best therapy for many people with hoarding difficulties is to be part of a normal community |  |  |  |  |  |

**Affiliate Stigma Scale**

Below are some sentences related to your own experience of having a close personal connection to somebody with OCD/Hoarding difficulties. We are asking how your being connected to the person you offer support to impacts you. There are no right or wrong answers. Please read each sentence carefully then choose the option which best represents your opinion.

|  | **Strongly**  **disagree** | **Disagree** | **Agree** | **Strongly**  **agree** |
| --- | --- | --- | --- | --- |
| I feel inferior because I have a close personal connection to them. | ① | ② | ③ | ④ |
| I avoid communicating with them. | ① | ② | ③ | ④ |
| Other people would discriminate against me if I was with them. | ① | ② | ③ | ④ |
| I feel emotionally disturbed because I have a close personal connection to them. | ① | ② | ③ | ④ |
| I do not dare to tell others that I have a close personal connection to them. | ① | ② | ③ | ④ |
| My reputation is damaged because I have a close personal connection to them. | ① | ② | ③ | ④ |
| Their behaviour is embarrassing. | ① | ② | ③ | ④ |
| I avoid going out with them. | ① | ② | ③ | ④ |
| People’s attitudes towards me are negative when I am with them. | ① | ② | ③ | ④ |
| I feel helpless about having a close personal connection to them. | ① | ② | ③ | ④ |
| I reduce contact with my friends and relatives because I have a close personal connection to them. | ① | ② | ③ | ④ |
| Having a close personal connection to them has a negative impact on me. | ① | ② | ③ | ④ |
| I feel sad because I have a close personal connection to them. | ① | ② | ③ | ④ |
|  | **Strongly**  **disagree** | **Disagree** | **Agree** | **Strongly**  **agree** |
| When I am with them, I keep a relatively low profile. | ① | ② | ③ | ④ |
| Having a close personal connection to them makes me think that I am incompetent compared to other people. | ① | ② | ③ | ④ |
| I worry that other people will find out I have a close personal connection to them. | ① | ② | ③ | ④ |
| I reduce interacting with them. | ① | ② | ③ | ④ |
| Having a close personal connection to them makes me think that I am less than others. | ① | ② | ③ | ④ |
| I feel that I am under great pressure because I have a close personal connection to them. | ① | ② | ③ | ④ |
| I do not dare to participate in activities related to mental health lest other people suspect I have a close personal connection to them. | ① | ② | ③ | ④ |
| Having close personal connection to them makes me lose face. | ① | ② | ③ | ④ |
| I reduce contact with my neighbours because I have a close personal connection to them. | ① | ② | ③ | ④ |

**Burden Scale for Family Caregivers**

We are asking you for information about times when you are offering support to your family member or friend or close personal connection. The following statements often refer to the type of your assistance. Please indicate the best description for how things are when you are offering support.

|  | **Strongly agree** | **Agree** | **Disagree** | **Strongly disagree** |
| --- | --- | --- | --- | --- |
| My life satisfaction has suffered because of offering support. |  |  |  |  |
| From time to time I wish I could “run away” from the situation I am in. |  |  |  |  |
| Sometimes I feel that the person I am offering support to is using me. |  |  |  |  |
| It’s easy for me offering support. |  |  |  |  |
| The support I offer is acknowledged by the person I support. |  |  |  |  |
| The support I offer is acknowledged by others. |  |  |  |  |
| I feel like I am being forced into offering support. |  |  |  |  |
| The wishes of the person I am offering support to are reasonable in my opinion. |  |  |  |  |
| I have had to give up plans because of offering support. |  |  |  |  |
| Offering support takes a lot of my own strength. |  |  |  |  |
| I feel torn between the demands of my environment (such as family) and the demands of offering support. |  |  |  |  |
| I feel I have a good relationship with the person I am offering support to. |  |  |  |  |
| I have problems with other family members due to offering support. |  |  |  |  |
| I am worried about my future because of the support I offer. |  |  |  |  |
| I feel sad because of the fate of the person I am offering support to. |  |  |  |  |
| I can take care of other daily obligations to my satisfaction in addition to offering support. |  |  |  |  |

**Free text**

Would you like to write anything about your experience of offering support to somebody with OCD and/or hoarding difficulties? Please ensure your answer fits into the box below. If you do not wish to write anything, please enter 'N/a'.

We thank you for your time spent taking this survey.

This study aims to better understand the support that is being offered to people with hoarding difficulties and OCD. Finding out more about this will help plan more effective treatments and how best to involve those who support them.

We know that it can be difficult to offer support to somebody with these conditions.

If you would like to find out more information about OCD, please see this helpful website: <https://ocdaction.org.uk/> OCD Action also offer group support sessions via Zoom for parents or partners of people with OCD, more details about this can be found on the website.

If you would like to find out more information about hoarding difficulties, please see this helpful website: <https://hoardinguk.org/> Hoarding UK also provides information about support groups for people with hoarding difficulties and some tips for how family members can support them, more details about this can be found on the website.

Once the study has been completed, information about the findings will be made available via the OXICPTR website.

## Supplementary Information E: P-NSSQ-R Items and Scoring Guide

**Adapting Norbeck for carer self-report**

- Relationship
  - What is your relationship to the person?
    1. Spouse or partner
    2. Significant other
    3. Family member or relative
    4. Neighbour
    5. Friend
    6. Work associate
    7. Health care provider
    8. Counsellor or therapist
    9. Minister/Priest/Rabbi/Imam
    10. Other (please specify…)
  - How long have you known this person (remove Likert, use full range)
- Contact items (**all are reverse coded**)
  - 1) How frequently do you usually attempt to make contact with this person via phone call/letter/email/social media? (4 = daily/3 = weekly/2 = monthly/1 = A few times a year/0 = once a year or less)
  - 2) How often does the person usually respond? (4 = every time/3 = most of the time/2 = half the time or more/1 = less than half the time/0 = rarely or never)
  - 3) How frequently does the person usually attempt to make contact with you via phone call/letter/email/social media? (4 = daily/3 = weekly/2 = monthly/1 = A few times a year/0 = once a year or less)
  - 4) How often do you usually respond? (4 = every time/3 = most of the time/2 = half the time or more/1 = less than half the time/0 = rarely or never)
  - 5) How frequently do you usually see this person face-to-face at your home or in a public place? (4 = daily/3 = weekly/2 = monthly/1 = A few times a year/0 = once a year or less)
  - 6) How frequently do you usually see this person in their home? (4 = daily/3 = weekly/2 = monthly/1 = A few times a year/0 = once a year or less)
  - 7) When was your most recent contact with this person? (4 = yesterday/3 = last week/2 = last month/1 = A few times a year/0 = once a year or less)

Following items 5-point Likert: 0 = not at all/1 = a little/2 = moderately/3 = quite a bit/4 = a great deal

Reversed items in **bold**

- Emotional support
  - Affect
    - How much do you try to make the person feel liked or loved?
    - How much do you actually make this person feel liked or loved?
    - How much do you try to make this person feel respected or admired?
    - How much do you actually make this person feel respected or admired?
  - Affirmation

1. How open are you to this person confiding in you?
2. How much does the person confide in you?
3. **How much do you agree with or support this person’s actions or thoughts regarding over acquiring and keeping possessions and/or obsessional and compulsive problems?**
4. How much do you agree with or support this person’s actions or thoughts (other than matters regarding over acquiring and keeping possessions and/or obsessional and compulsive problems)?

- Tangible support
  - Routine support
    - How available are you to help and support the person to do what they need to do?
    - How much does the person accept help and support from you with doing what they need to do?
    - How available are you to help and support the person meet their basic needs?
    - How much does the person accept help and support from you with meeting their basic needs?
  - Additional support

1. If the person needed help and support for several weeks, how available could you be to help this person?
2. How much would the person accept help and support from you if you offered this for several weeks?
3. If the person needed help and support in an emergency, how available could you usually be?
4. How much would the person accept help and support from you in an emergency?

- Perceived effectiveness
  - PWC response
    - How warmly does the person respond to your efforts to help and support them?
    - Is the person grateful of your efforts to help and support them?
    - Does the person recognise the effort you put into helping and supporting them?
    - Is the person welcoming of your help and support?
    - **Does the person reject your help and support?**
    - **How pointless does it feel to offer help and support to the person?**
    - How confident are you that you are offering the right kind of help and support to the person?
    - Does the help and support you offer to the person make a difference?
  - POS response

1. **How frustrated do you feel when offering help and support to the person?**
2. **How often do you snap at the person when offering help and support?**
3. How satisfied are you that you do your best to help and support to the person?
4. **Do you feel hopeless about offering help and support to the person?**
5. **Do you get angry with the person when offering help and support?**
6. Do you find helping and supporting the person rewarding?
7. **Do you feel resentful towards the person that you help and support?**
8. **Do you feel guilty about not offering enough help and support to the person?**

- Reciprocity (POS rating of support from PWC in turn)
  - Emotional support
    1. How much does this person make you feel liked or loved?
    2. How much does this person make you feel respected or admired?
    3. How much can you confide in this person?
    4. How much does this person agree with or support your actions or thoughts (other than matters regarding over acquiring and keeping possessions and/or obsessional and compulsive problems)?
  - Tangible support

1. If you needed immediate help, how much could this person usually help?
2. If you needed help for several weeks, how much could this person help you?

Calculating subscales

*Wish to support* = sum of 8 items:

- Emotional support items 1, 3, 5
- Tangible support items 1, 3, 5, 7
- Perceived effectiveness item 11

*Perceived success of support* = sum of 13 items:

- Emotional support items 2, 4, 6
- Tangible support items 2, 4, 6, 8
- Perceived effectiveness items 1-5, 8

*Impact of support on POS* = sum of 9 items:

- Perceived effectiveness items 6, 7, 9, 10, 12-16

*Contact* = sum of 7 contact items

*Reciprocity* = sum of 6 reciprocity items

## Supplementary Information F: P-NSSQ-R pilot and reliability analysis

Internal consistency of the P-NSSQ-R subscales related to the main hypothesis was checked at an initial pilot phase. Seven responses from POS(OCD) and five from POS(HD) were analysed and Cronbach’s alpha ranged from good to excellent, with the removal of any single item not producing an improved rating for any of the scales (wish to support α=.73, perceived support success α=.90, impact of support on POS α=.84). These respondents were invited to provide feedback on the survey and consequently two standard inverted NSSQ items were omitted from subsequent analyses on the basis that multiple respondents found the items confusing (‘how much do you agree with or support this person’s actions or thoughts regarding over acquiring and keeping possessions and/or obsessional and compulsive problems?’ and ‘how much do you agree with or support this person’s actions or thoughts (other than matters regarding over acquiring and keeping possessions and/or obsessional and compulsive problems)?’). Internal consistency was re-checked and was maintained or improved in the full sample (wish to support α=.83, perceived support success α=.94, impact of support on POS α=.83).

Due to using abridged versions for the original scales, the internal consistency was also checked for the CAMI (OCD α=.66, HD α=.71) and BSFC (α=.89), indicating acceptable to good reliability. Internal consistency for the ASS was rated as excellent (α=.95).

## Supplementary Information G: Full demographics table

|  | **Group** | |  |
| --- | --- | --- | --- |
|  | HD  (*N* = 69) | OCD  (*N* = 42) | Total  (*N* = 111) |
| **Relationship between POS and PWC** | | | |
| Relationship type |  |  |  |
| Spouse or partner | 8 (11.6%) | 16 (38.1%) | 24 (21.6%) |
| Significant other | 5 (7.2%) | 2 (4.8%) | 7 (6.3%) |
| Family member or relative | 36 (52.2) | 16 (38.1%) | 52 (46.8%) |
| Neighbour | 1 (1.4%) | - | 1 (.9%) |
| Friend | 18 (26.1%) | 5 (11.9%) | 23 (20.7%) |
| Work associate | - | 1 (2.4%) | 1 (.9%) |
| Other | 1 (1.4%) | 2 (4.8%) | 3 (2.7%) |
| Years known | *M* = 32.4, *SD* = 17.4 | *M* = 22.5, *SD* = 16.4 | *M* = 28.7, *SD* = 17.1 |
| Have you ever lived with the PWC? |  |  |  |
| Currently, for more than 10 years | 14 (20.3%) | 13 (31.0%) | 27 (24.3%) |
| Currently, for less than 10 years | 6 (8.7%) | 12 (28.6%) | 18 (16.2%) |
| Previously, for more than 10 years | 18 (26.1%) | 8 (19.0%) | 26 (23.4%) |
| Previously, for less than 10 years | 5 (7.2%) | 1 (2.4%) | 6 (5.4%) |
| Never lived together | 26 (37.7%) | 8 (19.0%) | 34 (30.6%) |
| **PWC demographics** | | | |
| Formal Diagnoses |  |  |  |
| Hoarding Disorder | 6 (8.7%) | - | 6 (5.4%) |
| Obsessive Compulsive Disorder | 3 (4.3%) | 29 (69.0%) | 32 (28.8%) |
| Hoarding Disorder and Obsessive Compulsive Disorder | - | 1 (2.4%) | 1 (.9%) |
| No formal diagnosis of Hoarding Disorder nor Obsessive Compulsive Disorder | 60 (87.0%) | 12 (28.6%) | 72 (64.9%) |
| Has the PWC sought help for their condition? |  |  |  |
| Yes | 18 (26.1%) | 31 (73.8%) | 49 (44.1%) |
| No | 51 (73.9%) | 11 (26.2 %) | 62 (55.9%) |
| Years since onset of condition | *M* = 25.6, *SD* = 13.7 | *M* = 22.2, *SD* = 11.9 | *M* = 24.3, *SD* = 13.1 |
| Age | *M* = 63.0, *SD* = 12.9 | *M* = 41.2, *SD* = 10.7 | *M* = 54.8, *SD* = 16.1 |
| Gender identity |  |  |  |
| Woman | 39 (56.5%) | 21 (50.0%) | 60 (54.1%) |
| Man | 28 (40.6%) | 19 (45.2%) | 47 (42.3%) |
| Transgender | 2 (2.9%) | - | 2 (1.8%) |
| Non-binary | - | 2 (4.8%) | 2 (1.8%) |
| Ethnicity |  |  |  |
| White (English / Welsh / Scottish / Northern Irish / British) | 59 (85%) | 35 (83.3%) | 94 (84.7%) |
| White (Irish) | 1 (1.4%) | - | 1 (.9%) |
| White (Any other White Background) | 4 (5.8%) | 2 (4.8%) | 6 (5.4%) |
| Mixed / Multiple Ethnic Groups (White and Black Caribbean) | 1 (1.4%) | 1 (2.4%) | 2 (1.8%) |
| Mixed / Multiple Ethnic Groups (White and Asian) | - | 2 (4.8%) | 2 (1.8%) |
| Mixed / Multiple Ethnic Groups (Any other Mixed / Multiple Ethnic Background) | - | 1 (2.4%) | 1 (.9%) |
| Asian / Asian British (Indian) | 1 (1.4%) | - | 1 (.9%) |
| Asian / Asian British (Pakistani) | 1 (1.4%) | 1 (2.4%) | 2 (1.8%) |
| Asian / Asian British (Any other Asian Background) | 1 (1.4%) | - | 1 (.9%) |
| Black / African / Caribbean / Black British (Any other Black / African / Caribbean Background) | 1 (1.4%) | - | 1 (.9%) |
| Marital status |  |  |  |
| Married or in a civil partnership | 18 (26.1) | 11 (26.2%) | 29 (26.1%) |
| Cohabiting | 7 (10.1%) | 8 (19.0%) | 15 (13.5%) |
| Divorced or separated | 12 (17.4%) | 1 (2.4%) | 13 (11.7%) |
| Widowed | 7 (10.1%) | 1 (2.4%) | 8 (7.2%) |
| Single | 25 (36.2%) | 21 (50.0%) | 46 (41.4%) |
| Employment status |  |  |  |
| Employed full-time | 12 (17.4%) | 15 (35.7%) | 27 (24.3%) |
| Employed part-time | 10 (14.5%) | 6 (14.3%) | 16 (14.4%) |
| Unemployed | 14 (20.3%) | 16 (38.1%) | 30 (27.0%) |
| Retired | 33 (47.8%) | 4 (9.5%) | 37 (33.3%) |
| Volunteering | - | 1 (2.4%) | 1 (.9%) |
| Highest educational attainment |  |  |  |
| No formal Qualification | 10 (14.5%) | 5 (11.9%) | 15 (13.5%) |
| GCSEs/GNVQs/Scottish Standard | 11 (15.9%) | 11 (26.2%) | 22 (19.8%) |
| A-Levels/BTEC/Scottish Higher | 12 (17.4%) | 4 (9.5%) | 16 (14.4%) |
| Apprenticeship | 4 (5.8%) | - | 4 (3.6%) |
| Graduate Degree (e.g. BA, BSc) | 21 (30.4%) | 9 (21.4%) | 30 (27%) |
| Higher Degree (e.g. MA, MSc, PGCE) | 8 (11.6%) | 10 (23.8%) | 18 (16.2%) |
| Doctorate (e.g. PhD, EdD) | 1 (1.4%) | 2 (4.8%) | 3 (2.7%) |
| Prefer not to say | 2 (2.9%) | 1 (2.4%) | 3 (2.7%) |
| **POS demographics** | | | |
| Age | *M* = 52.2, *SD* = 13.4 | *M* = 47.6, *SD* = 14.6 | *M* = 50.4, *SD* = 14.0 |
| Gender identity |  |  |  |
| Woman | 59 (85.5%) | 28 (66.7%) | 87 (78.4%) |
| Man | 6 (8.7%) | 12 (28.6%) | 18 (16.2%) |
| Non-binary | 3 (4.3%) | 1 (2.4%) | 4 (3.6%) |
| Prefer not to say | 1 (1.4%) | 1 (2.4%) | 2 (1.8%) |
| Ethnicity |  |  |  |
| White (English / Welsh / Scottish / Northern Irish / British) | 60 (87.0%) | 33 (78.6%) | 93 (83.8%) |
| White (Irish) | 1 (1.4%) | - | 1 (.9%) |
| White (Any other White Background) | 2 (2.9%) | 5 (11.9%) | 7 (6.3%) |
| Mixed / Multiple Ethnic Groups (White and Black Caribbean) | - | 1 (2.4%) | 1 (.9%) |
| Mixed / Multiple Ethnic Groups (White and Asian) | - | 1 (2.4%) | 1 (.9%) |
| Mixed / Multiple Ethnic Groups (Any other Mixed / Multiple Ethnic Background) | 2 (2.9%) | - | 2 (1.8%) |
| Asian / Asian British (Indian) | 1 (1.4%) | - | 1 (.9%) |
| Asian / Asian British (Pakistani) | 1 (1.4%) | 1 (2.4%) | 2 (1.8%) |
| Asian / Asian British (Any other Asian Background) | 1 (1.4%) | 1 (2.4%) | 2 (1.8%) |
| Black / African / Caribbean / Black British (Any other Black / African / Caribbean Background) | 1 (1.4%) | - | 1 (.9%) |
| Marital status |  |  |  |
| Married or in a civil partnership | 30 (43.5%) | 18 (42.9%) | 48 (43.2%) |
| Cohabiting | 15 (21.7%) | 11 (26.2%) | 26 (23.4%) |
| Divorced or separated | 8 (11.6%) | 2 (4.8%) | 10 (9.0%) |
| Widowed | - | 3 (7.1%) | 3 (2.7%) |
| Single | 16 (23.2%) | 8 (19.0%) | 24 (21.6%) |
| Employment status |  |  |  |
| Employed full-time | 29 (42.0%) | 17 (40.5%) | 46 (41.4%) |
| Employed part-time | 15 (21.7%) | 10 (23.8%) | 25 (22.5%) |
| Unemployed | 4 (5.8%) | 4 (9.5%) | 8 (7.2%) |
| Retired | 17 (24.6%) | 8 (19.0%) | 25 (22.5%) |
| Volunteering | 4 (5.8%) | 3 (7.1%) | 7 (6.3%) |
| Highest educational attainment |  |  |  |
| No formal Qualification | 2 (2.9%) | 4 (9.5%) | 6 (5.4%) |
| GCSEs/GNVQs/Scottish Standard | 4 (5.8%) | 5 (11.9%) | 9 (8.1%) |
| A-Levels/BTEC/Scottish Higher | 15 (21.7%) | 2 (4.8%) | 17 (15.3%) |
| Apprenticeship | - | 1 (2.4%) | 1 (.9%) |
| Graduate Degree (e.g. BA, BSc) | 27 (39.1%) | 19 (45.2%) | 46 (41.4%) |
| Higher Degree (e.g. MA, MSc, PGCE) | 17 (24.6%) | 7 (16.7%) | 24 (21.6%) |
| Doctorate (e.g. PhD, EdD) | 3 (4.3%) | 3 (7.1%) | 6 (5.4%) |
| Prefer not to say | 1 (1.4%) | 1 (2.4%) | 2 (1.8%) |

## Supplementary Information H: Adapted Norbeck Full Scores

*(reverse coded items have been transformed)*

|  |  | HD (*n*=69) | | OCD (*n*=42) | |
| --- | --- | --- | --- | --- | --- |
| Item ID | Item as worded on survey | Mean | Standard Deviation | Mean | Standard Deviation |
| Contact 1 | How frequently do you usually attempt to make contact with this person via phone call/letter/email/social media? | 3.2174 | 1.02713 | 3.4762 | 1.04153 |
| Contact 2 | How often does the person usually respond? | 2.7681 | 1.12649 | 3.1429 | 1.09481 |
| Contact 3 | How frequently does the person usually attempt to make contact with you via phone call/letter/email/social media? | 2.6087 | 1.36357 | 3.2619 | 1.16994 |
| Contact 4 | How often do you usually respond? | 3.5942 | .71371 | 3.8095 | .67130 |
| Contact 5 | How frequently do you usually see this person face-to-face at your home or in a public place? | 2.5507 | 1.41978 | 3.1429 | 1.35379 |
| Contact 6 | How frequently do you usually see this person in their home? | 2.0580 | 1.59857 | 3.1190 | 1.41770 |
| Contact 7 | When was your most recent contact with this person? | 3.2319 | 1.07300 | 3.7381 | .82815 |
| Emotional support 1 | How much do you try to make the person feel liked or loved? | 3.0145 | 1.03601 | 3.5476 | .83235 |
| Emotional support 2 | How much do you actually make this person feel liked or loved? | 2.2174 | 1.09638 | 2.8810 | .94230 |
| Emotional support 3 | How much do you try to make this person feel respected or admired? | 2.5362 | 1.24357 | 3.2381 | 1.00752 |
| Emotional support 4 | How much do you actually make this person feel respected or admired? | 1.7971 | 1.21969 | 2.5238 | 1.01784 |
| Emotional support 5 | How open are you to this person confiding in you? | 3.2174 | 1.12288 | 3.7381 | .62701 |
| Emotional support 6 | How much does the person confide in you? | 2.3188 | 1.33387 | 2.8333 | 1.01011 |
| Emotional support 7 | How much do you agree with or support this person’s actions or thoughts regarding over acquiring and keeping possessions and/or obsessional and compulsive problems? | 2.8986 | 1.03106 | 2.3571 | 1.18572 |
| Emotional support 8 | How much do you agree with or support this person’s actions or thoughts (other than matters regarding over acquiring and keeping possessions and/or obsessional and compulsive problems)? | 1.9710 | 1.15008 | 2.4048 | 1.08334 |
| Tangible support 1 | How available are you to help and support the person to do what they need to do? | 3.0435 | .99165 | 3.3333 | .75439 |
| Tangible support 2 | How much does the person accept help and support from you with doing what they need to do? | 1.4783 | 1.23203 | 2.4762 | 1.27333 |
| Tangible support 3 | How available are you to help and support the person meet their basic needs? | 2.5942 | 1.15452 | 3.1429 | .97709 |
| Tangible support 4 | How much does the person accept help and support from you with meeting their basic needs? | 1.7246 | 1.34912 | 2.4048 | 1.34454 |
| Tangible support 5 | If the person needed help and support for several weeks, how available could you be to help this person? | 2.8551 | 1.04706 | 3.2381 | .95788 |
| Tangible support 6 | How much would the person accept help and support from you if you offered this for several weeks? | 1.6667 | 1.29099 | 2.7143 | 1.27424 |
| Tangible support 7 | If the person needed help and support in an emergency, how available could you usually be? | 3.5507 | .77718 | 3.7143 | .67302 |
| Tangible support 8 | How much would the person accept help and support from you in an emergency? | 2.6232 | 1.36232 | 3.3333 | 1.00406 |
| Perceived effectiveness 1 | How warmly does the person respond to your efforts to help and support them? | 1.7826 | 1.19889 | 2.5238 | 1.06469 |
| Perceived effectiveness 2 | Is the person grateful of your efforts to help and support them? | 1.8116 | 1.45799 | 2.6429 | 1.28446 |
| Perceived effectiveness 3 | Does the person recognise the effort you put into helping and supporting them? | 1.5507 | 1.36701 | 2.5238 | 1.34777 |
| Perceived effectiveness 4 | Is the person welcoming of your help and support? | 1.6377 | 1.33913 | 2.5000 | 1.21475 |
| Perceived effectiveness 5 | Does the person reject your help and support? | 2.1304 | 1.42368 | 2.5714 | 1.03930 |
| Perceived effectiveness 6 | How pointless does it feel to offer help and support to the person? | 1.6812 | 1.50970 | 2.0476 | 1.43054 |
| Perceived effectiveness 7 | How confident are you that you are offering the right kind of help and support to the person? | 1.6812 | 1.15673 | 1.7857 | 1.07149 |
| Perceived effectiveness 8 | Does the help and support you offer to the person make a difference? | 1.3188 | 1.24254 | 2.2619 | 1.16994 |
| Perceived effectiveness 9 | How frustrated do you feel when offering help and support to the person? | 1.2899 | 1.24972 | 1.8810 | 1.13056 |
| Perceived effectiveness 10 | How often do you snap at the person when offering help and support? | 2.8551 | 1.23996 | 2.8333 | 1.08012 |
| Perceived effectiveness 11 | How satisfied are you that you do your best to help and support to the person? | 2.5072 | 1.20810 | 2.4286 | 1.30931 |
| Perceived effectiveness 12 | Do you feel hopeless about offering help and support to the person? | 1.4783 | 1.35699 | 1.7857 | 1.35315 |
| Perceived effectiveness 13 | Do you get angry with the person when offering help and support? | 2.5797 | 1.45931 | 2.8095 | 1.17366 |
| Perceived effectiveness 14 | Do you find helping and supporting the person rewarding? | 1.3478 | 1.25822 | 2.0714 | 1.27629 |
| Perceived effectiveness 15 | Do you feel resentful towards the person that you help and support? | 2.5072 | 1.56828 | 3.1667 | 1.18767 |
| Perceived effectiveness 16 | Do you feel guilty about not offering enough help and support to the person? | 2.4783 | 1.41014 | 2.0238 | 1.38789 |
| Reciprocity 1 | How much does this person make you feel liked or loved? | 2.0000 | 1.26025 | 2.9762 | 1.19935 |
| Reciprocity 2 | How much does this person make you feel respected or admired? | 1.7971 | 1.31261 | 2.8333 | 1.36000 |
| Reciprocity 3 | How much can you confide in this person? | 1.7681 | 1.42607 | 2.8095 | 1.36575 |
| Reciprocity 4 | How much does this person agree with or support your actions or thoughts (other than matters regarding over acquiring and keeping possessions and/or obsessional and compulsive problems)? | 1.9420 | 1.13609 | 2.3571 | 1.20611 |
| Reciprocity 5 | If you needed immediate help, how much could this person usually help? | 2.0580 | 1.43367 | 2.5000 | 1.32977 |
| Reciprocity 6 | If you needed help for several weeks, how much could this person help you? | 1.7826 | 1.42323 | 2.2619 | 1.36256 |

## Supplementary Information I: Full description of supplementary analysis with grouping excluding comorbid OCD and HD

The primary and secondary analyses were re-run, omitting cases where the POS identified the PWC as having comorbid OCD or HD. In terms of group differences, results were broadly in line with the main group classification, however there were some discrepancies. With the ‘pure’ condition groups, there was a significant difference in POS age, the HD group being older: HD (*M*=51.7 *SD*=14.0), OCD (*M*=42.6, *SD*=13.5) *U*=379.5, *p*=011. There were also significantly more PWC currently unemployed in the HD group (64.0%) versus the OCD group (37.5%) *X*^2^ (1, *N*=74)=4.6, *p*=.032, and significantly more PWC in the HD group having a maximum educational attainment up to and including A-Levels (64.0%), compared to the OCD group (37.5%) *X*^2^ (1, *N* = 74)=4.6, *p*=.032. The grouping excluding comorbidity also showed that this HD group had a similar likelihood of having weekly face-to-face contact in the PWC homes to the OCD group *X*^2^ (1, *N* = 74)=2.2, *p*=.136 and a similar rate of the POS having ever lived with the PWC *X*^2^ (1, *N* = 74)=1.3, *p*=.246.

As per the first round of analysis, evaluation of the adapted NSSQ-R subscales found a significant main effect of subscale *F*_(2,144)_=60.0, *p*<.001 and a significant interaction effect of subscale x group *F*_(2,144)_=5.0, *p*=.008. One difference found was that the significant difference between perceived success and supporter experience subscales within the OCD group was no longer significant when removing comorbidity, *t*_(23)_=2.0, *p*=.063.

Running a mixed model for the ASS subscales between groups revealed a significant main effect of subscale *F*_(1,69)_=27.0, *p*<.001. Again, the interaction effect of subscale x group here was not significant *F*_(1,69)_=1.4, *p*=.237. There was a significant main effect of group *F*_(1,69)_=55.7, *p*<.001. For the CAMI, another significant main effect of subscale was detected *F*_(1,70)_=14.0, *p*<.001, with the interaction effect of subscale x group still not meeting significance *F*_(1,70)_=.25, *p*=.874. There was a difference from the first round of analysis in that the main effect of group was not significant *F*_(1,70)_=3.6, *p*=.062.
